# Diabetes Advances Cardiomyocyte Senescence Through Interfering *Rnd3* Expression and Function

**DOI:** 10.1111/acel.70031

**Published:** 2025-03-02

**Authors:** Linxu Wu, Xinglin Zhu, Shanshan Pan, Yan Chen, Cai Luo, Yangyang Zhao, Jingci Xing, Kaijia Shi, Shuya Zhang, Jiaqi Li, Jinxuan Chai, Xuebin Ling, Jianmin Qiu, Yan Wang, Zhihua Shen, Wei Jie, Junli Guo

**Affiliations:** ^1^ Key Laboratory of Tropical Translational Medicine of Ministry of Education & Hainan Provincial Key Laboratory for Tropical Cardiovascular Diseases Research, School of Public Health Hainan Medical University Haikou China; ^2^ Public Research Center of Hainan Medical University Haikou China; ^3^ Department of Pathology and Pathophysiology, School of Basic Medicine Sciences Guangdong Medical University Zhanjiang China; ^4^ Department of Cardiovascular Medicine The First Affiliated Hospital of Hainan Medical University Haikou China

**Keywords:** cardiomyocyte, cellular senescence, diabetic cardiomyopathy, miR‐103a‐3p, Rnd3, STAT3

## Abstract

Rnd3 is a small Rho‐GTPase that has been implicated in various cardiovascular diseases. Yet, its role in diabetes‐induced cardiomyocyte senescence remains unknown. Here we tested the role of Rnd3 in cardiomyocyte senescence and diabetic cardiomyopathy (DCM). The expression of *Rnd3* was found to be reduced in peripheral blood mononuclear cells from diabetic patients and correlated negatively with age but positively with cardiac function. In 96‐week‐old Sprague Dawley (SD) rats, cardiac function was impaired, accompanied by an increased number of SA‐β‐gal‐positive cells and elevated levels of the senescence‐associated secretory phenotype (SASP) related factors, compared to those of 12‐week‐old rats. Diabetes and high glucose (HG, 35 mmol/L D‐glucose) suppressed *Rnd3* expression in cardiomyocytes and induced cardiomyocyte senescence. The deficiency of *Rnd3* exacerbated cardiomyocyte senescence in vitro and in vivo. MicroRNA sequencing in AC16 cells identified a conserved miR‐103a‐3p (present in humans and rats) as a key HG‐upregulated microRNA that bound to the *Rnd3* 3′‐UTR. In cultured cardiomyocytes, miR‐103a‐3p inhibitors antagonized HG‐induced cardiomyocyte senescence dependent on *Rnd3* expression. Treatment with AAV9 vectors carrying miR‐103a‐3p sponges and *Rnd3*‐overexpressing plasmids alleviated cardiomyocyte senescence and restored cardiac function in diabetic SD rats. HG stimulation increased STAT3 (Tyr705) phosphorylation and promoted its nuclear translocation in H9C2 cells, an effect exacerbated by *Rnd3* knockout. Mechanistically, Rnd3 interacted with p‐STAT3 in the cytoplasm, facilitating proteasome‐mediated ubiquitination and p‐STAT3 degradation. The STAT3 inhibitor S3I‐201 blocked HG‐induced STAT3 activation and mitigated cardiomyocyte senescence. These findings suggest that diabetes induces cardiomyocyte senescence via the miR‐103a‐3p/Rnd3/STAT3 signaling pathway, highlighting a potential therapeutic target for DCM.

## Graphical abstract

Diabetes‐induced upregulation of miR‐103a‐3p suppresses *Rnd3* expression in cardiomyocytes, thereby promoting the senescence‐associated secretory phenotype (SASP) through activation of the STAT3 signaling pathway, mediated by UPS‐induced degradation of p‐STAT3. Intervention strategies—such as using an miR‐103a‐3p inhibitor/sponges, restoring *Rnd3* expression via AAV9, or a STAT3 inhibitor, effectively counteract diabetes‐induced cardiomyocyte senescence.

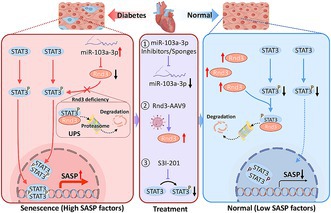

## 1 Introduction

The medical and social demographic challenges associated with global population aging are becoming increasingly significant (Centers for Disease and Prevention 2003). By 2050, it is estimated that 20% of the world's population will be over 65 years old (Dogra et al. 2022). This growth is occurring at an unprecedented rate and is expected to accelerate further, especially in developing countries. Concurrently, the prevalence of diabetes and other chronic diseases is also rising. In 2021, approximately 537 million adults worldwide were living with diabetes, and this number is projected to reach 783 million by 2045 (Sun et al. 2022). In the same year, diabetes was responsible for 6.7 million deaths. Research indicates a strong correlation between diabetes and aging, particularly among individuals over 65, where diabetes prevalence is as high as 25%–30% (American Diabetes 2018). Both aging and diabetes are independently recognized as major risk factors for cardiovascular disease (Dauriz et al. 2017; North and Sinclair 2012). Moreover, diabetes accelerates cellular senescence—commonly referred to as pathological senescence—and exacerbates age‐related cardiac damage through mechanisms such as fibrosis, disruptions in NAD+ metabolism, oxidative stress, and inhibition of autophagy (Abdellatif et al. 2021; Chen et al. 2022; Dewanjee et al. 2021; Jankauskas et al. 2021; Jia et al. 2018). Therefore, addressing diabetes‐induced cardiac cell senescence is critical for reducing the risk of cardiovascular events.

Senescent cardiomyocytes exhibit impaired contraction and metabolic dysfunction while secreting factors associated with the senescence‐associated secretory phenotype (SASP), which alters the local cardiac microenvironment (Chen et al. 2022; Tang et al. 2020). Although various mechanisms can induce cardiomyocyte senescence, metabolic stressors, such as diabetes, are key drivers of the senescent phenotype in cardiomyocytes (Tang et al. 2020). Unlike most other cell types, cardiomyocytes are terminally differentiated, meaning their senescence cannot be defined solely by cell cycle arrest. Instead, senescent cardiomyocytes are characterized by distinct features, including DNA damage, SASP expression, systolic dysfunction, endoplasmic reticulum stress, cellular hypertrophy, and mitochondrial dysfunction (Abdellatif et al. 2023; Cai et al. 2022; Ribeiro et al. 2023). While diabetes is a significant factor in promoting cardiomyocyte senescence, the underlying mechanisms and therapeutic targets, particularly under diabetic conditions, remain poorly understood.

Rnd3, also known as Rho family GTPase 3, is an atypical member of the Rho GTPase superfamily. It functions as an endogenous inhibitor of Rho‐associated kinase (ROCK) and plays a pivotal role in cytoskeletal organization, cell proliferation, migration, differentiation, cell cycle regulation, and apoptosis (Chardin 2006; Jie et al. 2015). Rnd3 is involved in several pathological processes, including brain edema caused by ependymal cell proliferation, stress‐induced cardiomyocyte apoptosis, chronic heart failure, post‐heart failure angiogenesis, arrhythmias, and post‐infarction inflammation (Dai et al. 2019; Lin et al. 2013; Yang et al. 2015; Yue et al. 2016, 2014). Moreover, studies have shown that cardiac fibroblast‐specific activation of Rnd3 has a protective effect in the pathogenesis of diabetic cardiomyopathy (DCM) (Zhang et al. 2022). Collectively, these findings suggest a protective role for Rnd3 in cardiovascular diseases. However, the relationship between Rnd3 and cardiomyocyte senescence, particularly in the context of diabetes, remains unclear.

MicroRNAs (miRNAs) are short RNA molecules, typically 21–23 nucleotides long, that regulate gene expression at the epigenetic level. Previous studies have reported that specific miRNAs can regulate Rnd3 (Jiang et al. 2020; Sun et al. 2015; Zhang et al. 2018). This study aimed to explore the mechanistic role of Rnd3 in cardiomyocyte senescence under diabetic conditions. Our findings reveal a novel role for miR‐103a‐3p‐mediated dysregulation of Rnd3 in diabetes‐induced cardiomyocyte senescence, offering a potential therapeutic strategy for DCM.

## 2 Results

### 2.1 *Rnd3* Expression in Human Tissues

**FIGURE 1:**
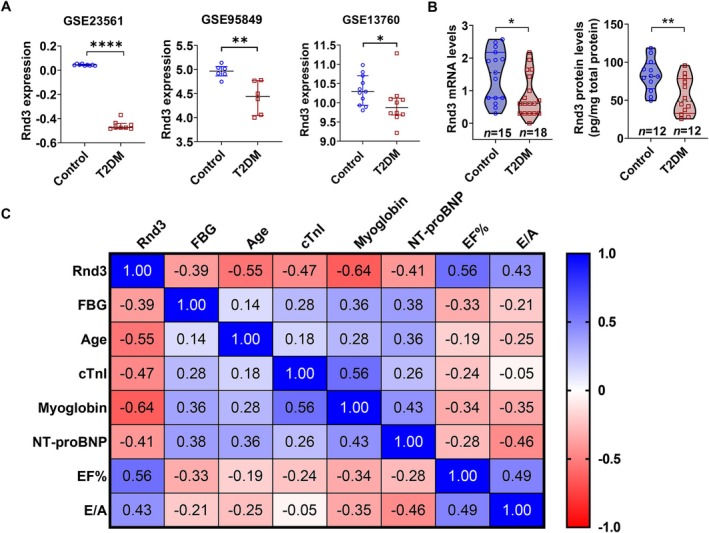
*Rnd3* expression in diabetes and control individuals. (A) gene expression omnibus (GEO) datasets show that *Rnd3* mRNA levels are significantly lower in peripheral blood cells (GSE23561, GSE95849) and coronary artery media (GSE13760) in diabetic patients compared to controls. Data were analyzed using the Mann–Whitney test; *p < 0.05, **p < 0.01, ****p < 0.0001. (B) *Rnd3* mRNA and protein levels in peripheral blood mononuclear cells from a clinical cohort of diabetic and nondiabetic patients. Data were analyzed using the Mann–Whitney test; *p < 0.05, **p < 0.01. (C) Spearman correlation analysis of *Rnd3* mRNA levels with fasting blood glucose (FBG), age, cardiac troponin I (cTnI), myoglobin, NT‐proBNP (N‐terminal pro‐brain natriuretic peptide), ejection fraction (EF), and E/A ratio (early‐to‐late diastolic transmitral flow velocity) in the clinical cohort.

To explore the relationship between *Rnd3* and diabetes, we analyzed type 2 diabetes mellitus (T2DM) data from the gene expression omnibus (GEO) database. *Rnd3* mRNA expression levels in peripheral blood mononuclear cells (GSE23561, GSE95849) and coronary tissue (GSE13760) were significantly lower in individuals with T2DM compared to nondiabetic controls (Figure 1A). In our cohort, *Rnd3* expression—both at the mRNA and protein levels—was also markedly reduced in the peripheral blood mononuclear cells of T2DM patients compared to controls (Figure 1B). Correlation analysis showed that *Rnd3* mRNA expression was significantly negatively correlated with age (*r* = −0.55, *p* = 0.001), fasting blood glucose (FBG, *r* = −0.39, *p* = 0.0497), cardiac troponin I (cTnI, *r* = −0.47, *p* = 0.0062), myoglobin (*r* = −0.64, *p* < 0.0001), and N‐terminal pro‐brain natriuretic peptide (NT‐proBNP, *r* = −0.41, *p* = 0.0224). Conversely, it was positively correlated with ejection fraction (EF, *r* = 0.56, *p* = 0.0007) and the early‐to‐late diastolic transmitral flow velocity (E/A ratio, *r* = 0.43, *p* = 0.036) (Figure 1C). For detailed characteristics of the clinical cohorts involved in *Rnd3* mRNA testing, refer to Table S1. These findings suggest that T2DM is associated with reduced *Rnd3* expression and that *Rnd3* mRNA levels are inversely related to aging and myocardial injury.

### 2.2 *Rnd3* Expression and Cardiac Function in Aged Rats

**FIGURE 2:**
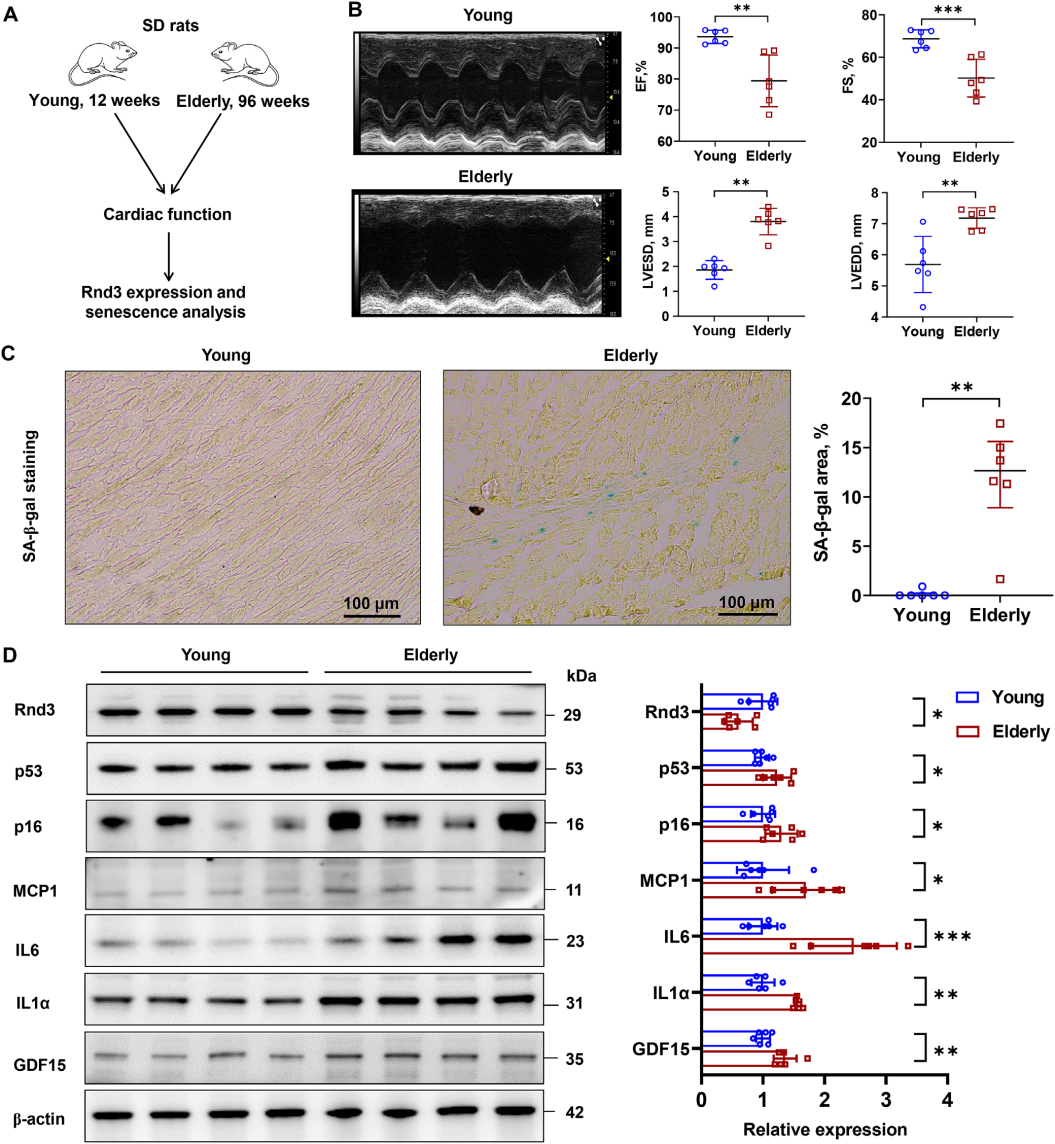
Rnd3 expression and cardiac function in adult and aged rats. (A) Schematic representation of the experimental procedure. (B) Ultrasound assessment of cardiac function in adult and aged rats. Data were analyzed using an unpaired t‐test; **p < 0.01, ***p < 0.001. (C) SA‐β‐galactosidase staining of frozen heart tissue sections. Data were analyzed using an unpaired *t*‐test; **p < 0.01. (D) Western blot analysis of Rnd3, cell cycle‐related proteins p53 and p16, and SASP‐related proteins IL‐1α, IL‐6, and GDF15 in heart tissue. β‐actin served as the internal reference. Data were analyzed using an unpaired *t*‐test; *p < 0.05, **p < 0.01, ***p < 0.001.

To investigate the effects of aging on *Rnd3* expression and cardiac function, we compared gene expression in heart tissue and cardiac function between adult and aged Sprague Dawley (SD) rats (Figure 2A). The EF and fractional shortening (FS) values in 96‐week‐old rats were significantly lower than those in 12‐week‐old rats, though still above the threshold for heart failure. Additionally, the left ventricular end‐systolic diameter (LVESD) and left ventricular end‐diastolic diameter (LVEDD) were significantly increased in the older rats (Figure 2B, Table S2). SA‐β‐gal‐positive cells were not detected in the heart tissue of 12‐week‐old rats, whereas a significant increase in SA‐β‐gal‐positive cells was observed in 96‐week‐old rats (Figure 2C). Western blot analysis revealed reduced Rnd3 protein levels in aged rats, accompanied by increased markers of cellular senescence, including p53, p16, monocyte chemoattractant protein‐1 (MCP1), interleukin‐6 (IL‐6), interleukin‐1α (IL‐1α), and growth differentiation factor 15 (GDF15) (Figure 2D).

### 2.3 Diabetes Inhibits *Rnd3* Expression in Heart Tissue and Induces Cardiomyocyte Senescence

**FIGURE 3:**
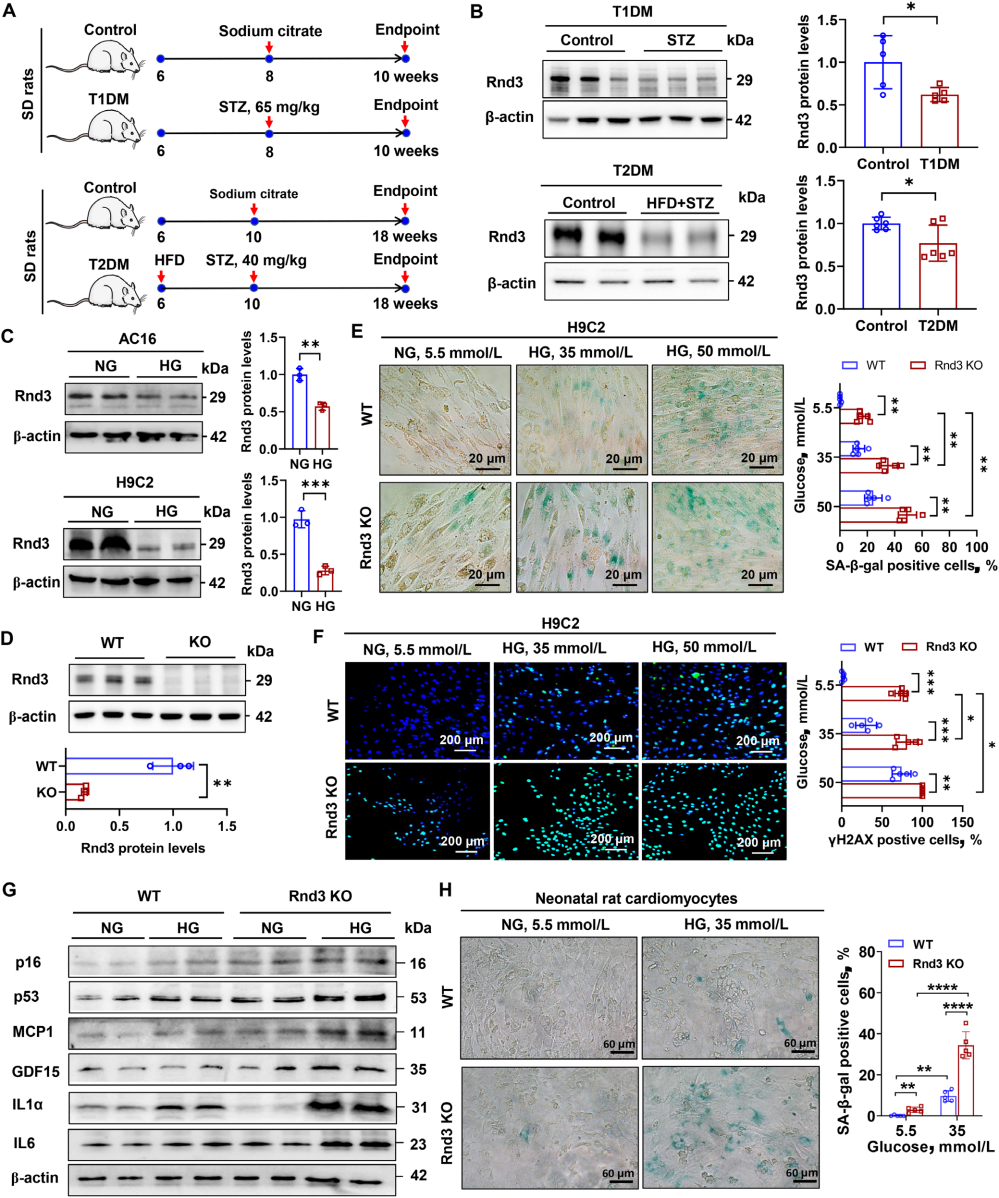
Effects of diabetes/high glucose on *Rnd3* expression and cell senescence in cardiomyocytes. (A) Schematic diagram of the establishment of T1DM and T2DM models. (B) Western blot analysis of Rnd3 protein levels in heart tissue of diabetic rats. β‐actin served as the internal reference. Data were analyzed using an unpaired *t*‐test; *n* = 5 or *n* = 6, *p < 0.05. (C) Western blot analysis of Rnd3 protein levels in high glucose (HG) (35 mmol/L D‐glucose)‐treated cardiomyocytes. Data were analyzed using an unpaired *t*‐test; *n* = 3, **p < 0.01, ***p < 0.001. (D) Generation of *Rnd3* knockout H9C2 cardiomyocytes using CRISPR/Cas9 technology. Data were analyzed using an unpaired *t*‐test; *n* = 3, **p < 0.01. (E) SA‐β‐galactosidase staining in normal glucose (NG)‐ and HG‐treated H9C2 cells. Data were analyzed using an unpaired *t*‐test; *n* = 5, **p < 0.01. (F) Immunofluorescence staining of γH2AX in NG (5.5 mmol/L D‐glucose)‐treated and HG (35 mmol/L D‐glucose)‐treated H9C2 cells. Data were analyzed using an unpaired *t*‐test; *n* = 5, *p < 0.05, **p < 0.01, ***p < 0.001. (G) Western blot analysis of cell cycle‐related proteins p53 and p16, and SASP‐related proteins MCP1, IL‐1α, IL‐6, and GDF15 in NG‐ and HG‐treated H9C2 cells. β‐actin served as the internal reference. (H) SA‐β‐galactosidase staining in cardiomyocytes of neonatal rats treated with NG (5.5 mmol/L D‐glucose) or HG (35 mmol/L D‐glucose). Data were analyzed using an unpaired t‐test; *n* = 5, **p < 0.05, ****p < 0.0001.

In streptozotocin (STZ)‐induced rat models of type 1 diabetes mellitus (T1DM) and T2DM (Figure 3A), Rnd3 expression in heart tissue was significantly lower than in controls (Figure 3B). For details of T1DM and T2DM rats, please see Tables S3 and S4. Given the high expression of *Rnd3* in normal heart tissue cardiomyocytes (Figure S1), we investigated the impact of diabetes on *Rnd3* expression in these cells. In vitro experiments revealed that high glucose (HG, 35 mmol/L D‐glucose) stimulation significantly suppressed *Rnd3* expression in cardiomyocytes (Figure 3C) in a glucose‐dependent manner (Figure S2a,b). Using CRISPR/Cas9, we constructed *Rnd3* knockout H9C2 cells (Figure 3D). HG stimulation significantly increased SA‐β‐gal‐positive staining in cardiomyocytes, an effect amplified by *Rnd3* knockout (Figure 3E). This was accompanied by aggravated DNA damage (Figure 3F) and upregulation of cell cycle arrest molecules (p53 and p16) and SASP‐related factors (MCP1, GDF15, IL‐1α, and IL‐6) (Figure 3G, Figure S3). Importantly, these effects were independent of osmotic pressure (Figure S2c–e). Similar results were observed in neonatal rat cardiomyocytes, where elevated glucose levels and *Rnd3* knockout synergistically accelerated cardiomyocyte senescence (Figure 3H).

**FIGURE 4:**
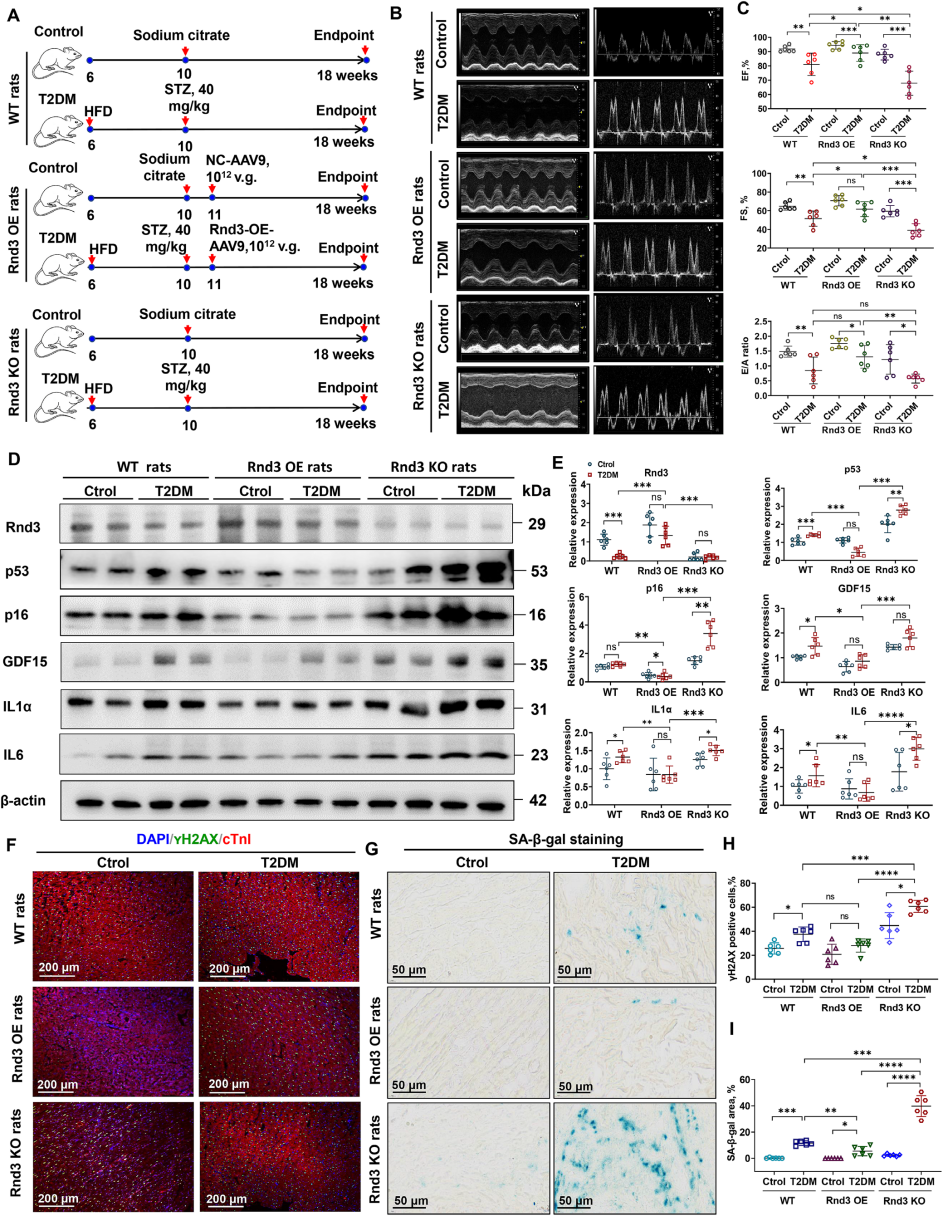
Cardiac function and cellular senescence analysis in rats with or without *Rnd3* gene‐targeted intervention. (A) Schematic diagram of the establishment of T2DM rats. (B) Representative images of echocardiography. (C) Cardiac function in rats with or without *Rnd3* gene intervention. Data were analyzed using an unpaired *t*‐test; *n* = 6, *p < 0.05, **p < 0.01, ***p < 0.001. ns, no significance. (D, E) Western blot analysis of Rnd3, cell cycle‐related proteins p53 and p16, and SASP‐related proteins IL‐1α, IL‐6, and GDF15 in rats with or without *Rnd3* gene intervention. β‐actin served as the internal reference. Data were analyzed using an unpaired *t*‐test; *n* = 6, *p < 0.05, **p < 0.01, ***p < 0.001, ****p < 0.0001. ns, no significance. (F, H) Immunofluorescence staining and quantitative analysis of γH2AX in heart tissue from rats with or without *Rnd3* gene intervention. Data were analyzed using an unpaired *t*‐test; *n* = 6, *p < 0.05, ***p < 0.001, ****p < 0.0001. ns, no significance. (G, I) SA‐β‐galactosidase staining in frozen heart sections of rats with or without *Rnd3* gene intervention. Data were analyzed using an unpaired *t*‐test; *n* = 6, *p < 0.05, **p < 0.01, ***p < 0.001, ****p < 0.0001. ns, no significance.

Considering the global prevalence of T2DM, which is higher than that of T1DM and more commonly seen in adults, our study focuses on exploring the functions and associated mechanisms of *Rnd3* using T2DM animal models. In vivo, we constructed T2DM rat models with cardiomyocyte‐specific *Rnd3* knockout (KO), overexpression (OE), and wild‐type (WT) controls (Figure 4A, Figure S4, Table S4). We excluded potential myocardial damage effects from αMHC^Cre+/+^ (Figure S5). Echocardiography revealed that T2DM reduced EF and FS and inverted the E/A ratio, with *Rnd3* KO exacerbating these changes and *Rnd3* OE mitigating them (Figure 4B,C). Western blot analysis showed that *Rnd3* KO increased senescence markers in diabetic heart tissue, whereas *Rnd3* OE reduced them (Figure 4D,E). γH2AX and SA‐β‐gal staining confirmed that *Rnd3* KO promoted senescence in diabetic myocardial cells, while *Rnd3* OE reversed these effects (Figure 4F–I).

### 2.4 HG Stimulation Upregulates miR‐103a‐3p to Inhibit *Rnd3* Expression in Cardiomyocytes

**FIGURE 5:**
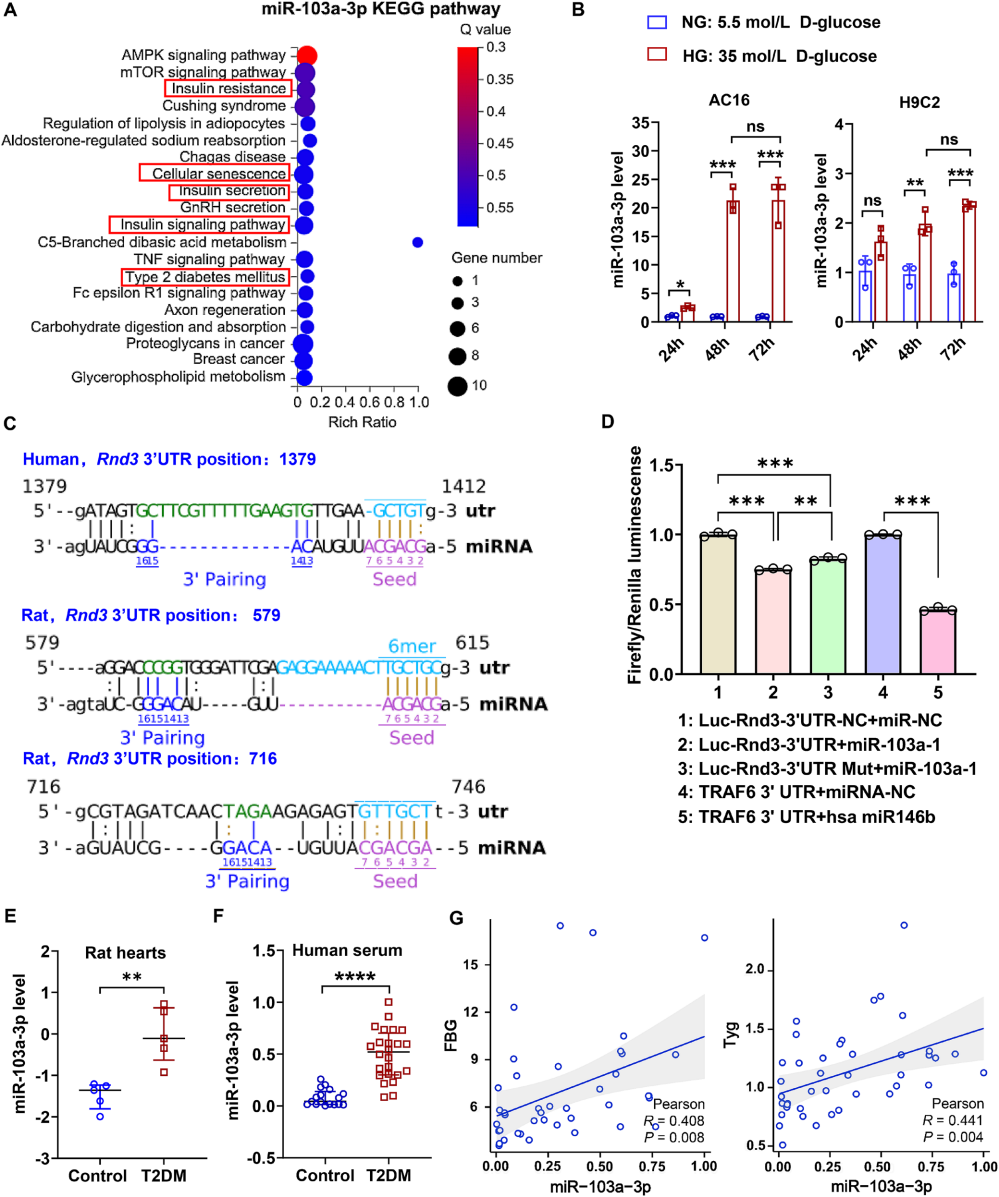
Function and expression of miR‐103a‐3p. (A) Kyoto Encyclopedia of Genes and Genomes (KEGG) analysis of miR‐103a‐3p. KEGG pathways in the red box indicate that miR‐103a‐3p is associated with cellular senescence and diabetes. (B) Quantitative RT‐PCR analysis of miR‐103a‐3p in NG (5.5 mmol/L D‐glucose)‐treated and HG (35 mmol/L D‐glucose)‐treated cardiomyocytes. Data were analyzed using an unpaired *t*‐test; *n* = 3, ns, none significance, *p < 0.05, **p < 0.01, ***p < 0.001. (C) Analysis of binding sites between miR‐103a‐3p and the 3′UTR of the human and rat *Rnd3* gene. (D) Dual luciferase reporter assay to detect the binding effect of miR‐103a‐3p with the rat *Rnd3* gene 3′UTR. Groups 4 and 5 serve as negative and positive controls to demonstrate the reliability of the experimental system. Data were analyzed using the Bonferroni post hoc test following one‐way analysis of variance; *n* = 3, **p < 0.01, ***p < 0.001. (E) Quantitative RT‐PCR analysis of miR‐103a‐3p in left ventricle tissues of T2DM (*n* = 5) and control (*n* = 5) rats. Data were analyzed using the unpaired *t*‐test; **p < 0.01. (F) Quantitative RT‐PCR analysis of miR‐103a‐3p in serum samples from a clinical cohort of T2DM (*n* = 24) and control (*n* = 17) individuals. Data were analyzed using the Mann–Whitney test; ****p < 0.0001. (G) Pearson correlation analysis of miR‐103a‐3p levels with fasting blood glucose (FBG) and triglyceride and glucose index (Tyg) in a clinical cohort (*n* = 41).

To identify miRNAs regulated by glucose that target *Rnd3*, we performed miRNA sequencing on AC16 cells treated with normal glucose (NG, 5.5 mmol/L) and HG (35 mmol/L). After 48 h of HG treatment, 43 differentially expressed miRNAs (23 upregulated, 20 downregulated) were identified (Table S5). We focused on upregulated miRNAs, as most inhibit target gene expression. Among these, miR‐103a‐3p was strongly linked to T2DM and cellular senescence (Figure 5A). Further experiments showed that miR‐103a‐3p expression was increased in HG‐stimulated cardiomyocytes in vitro (Figure 5B). Sequence alignment revealed that miR‐103a‐3p is highly conserved between humans and rats, with binding sites in the *Rnd3* 3′UTR (Figure 5C). Luciferase reporter assays confirmed that miR‐103a‐3p directly inhibits *Rnd3* expression (Figure 5D). Heart tissues from the T2DM rats displayed an upregulation of miR‐103a‐3p levels compared with control rats (Figure 5E). Clinical analysis of plasma samples from 24 T2DM patients and 17 controls (Table S6) showed significantly higher miR‐103a‐3p levels in the T2DM group (Figure 5F). miR‐103a‐3p levels were positively correlated with FBG (*r* = 0.408, *p* = 0.008) and the triglyceride and glucose index (Tyg, *r* = 0.441, *p* = 0.004) (Figure 5G) in humans.

### 2.5 Downregulating miR‐103a‐3p In Vitro Restores *Rnd3* Expression and Alleviates HG‐Induced Cardiomyocyte Senescence

**FIGURE 6:**
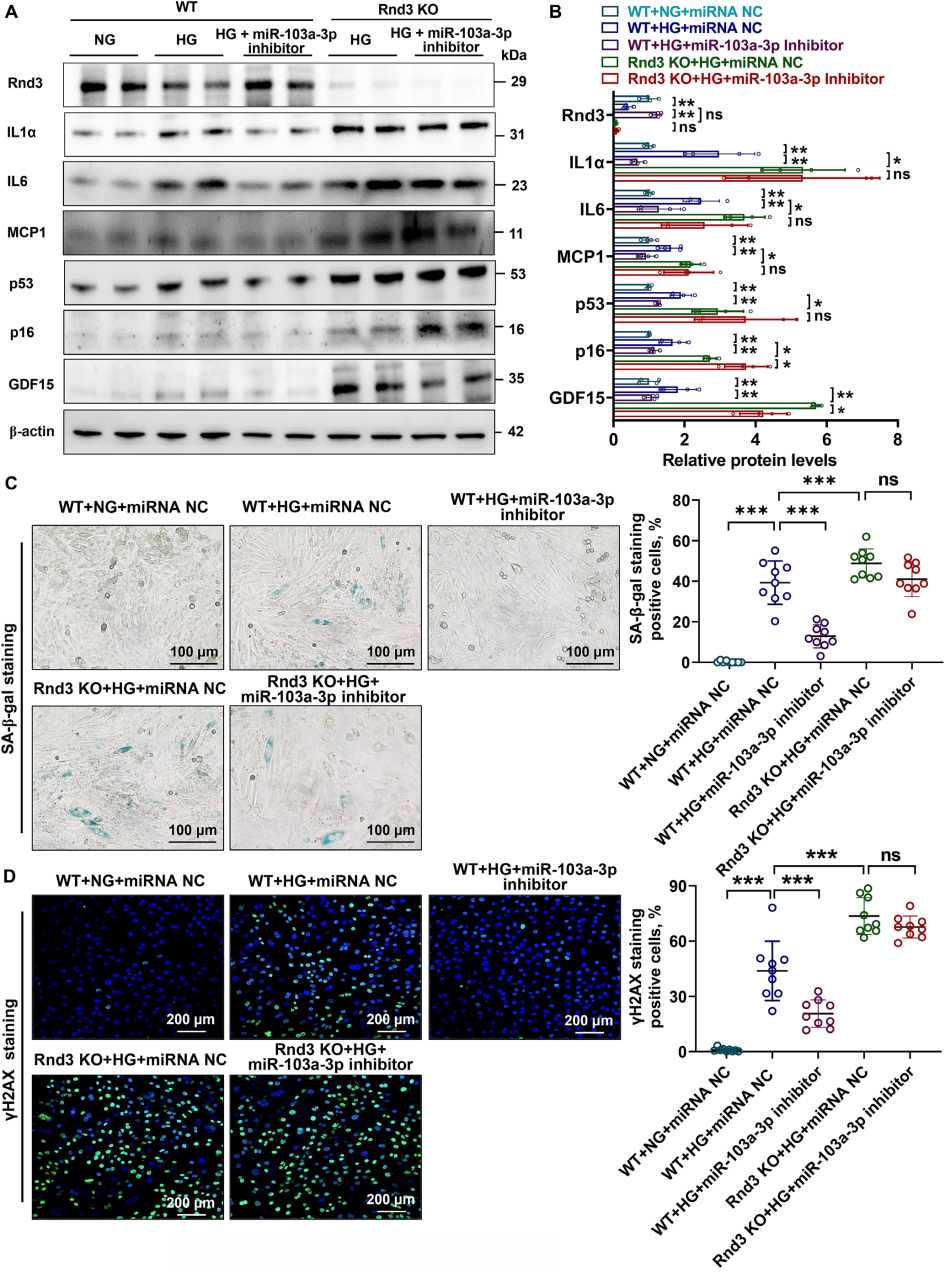
Effects of an miR‐103a‐3p inhibitor on Rnd3 expression and cardiomyocyte senescence. (A, B) Western blot analysis of Rnd3, cell cycle‐related proteins p53 and p16, and SASP‐related proteins MCP1, IL‐1α, IL‐6, and GDF15 in HG (35 mmol/L)‐treated H9C2 cells. β‐actin served as the internal reference. Data were analyzed using an unpaired *t*‐test; *n* = 4, *p < 0.05, **p < 0.01. ns, no significance. (C, D) SA‐β‐galactosidase and γH2AX staining in HG (35 mmol/L)‐treated H9C2 cells. Data were analyzed using an unpaired *t*‐test; *n* = 9, ***p < 0.001. ns, no significance.

To assess the therapeutic potential of targeting miR‐103a‐3p, we designed an miR‐103a‐3p inhibitor and applied it to HG‐stimulated H9C2 cells. Treatment with the inhibitor significantly increased *Rnd3* expression and reduced the expression of senescence‐related genes. However, *Rnd3* knockdown diminished the inhibitor's effects on SASP (Figure 6A,B, Figure S6). SA‐β‐gal and γH2AX staining confirmed that the inhibitor alleviated HG‐induced cardiomyocyte senescence and DNA damage, effects that were attenuated in *Rnd3* knockout cells (Figure 6C,D). The use of this inhibitor also significantly decreased cell senescence in HG‐induced AC16 (Figure S7). These findings highlight the dependency of miR‐103a‐3p inhibitors on *Rnd3* for their therapeutic effects, laying the groundwork for in vivo experiments.

### 2.6 Cardiac Overexpression of miR‐103a‐3p Sponges Restores *Rnd3* Expression and Improves Heart Function in Diabetic Rats

**FIGURE 7:**
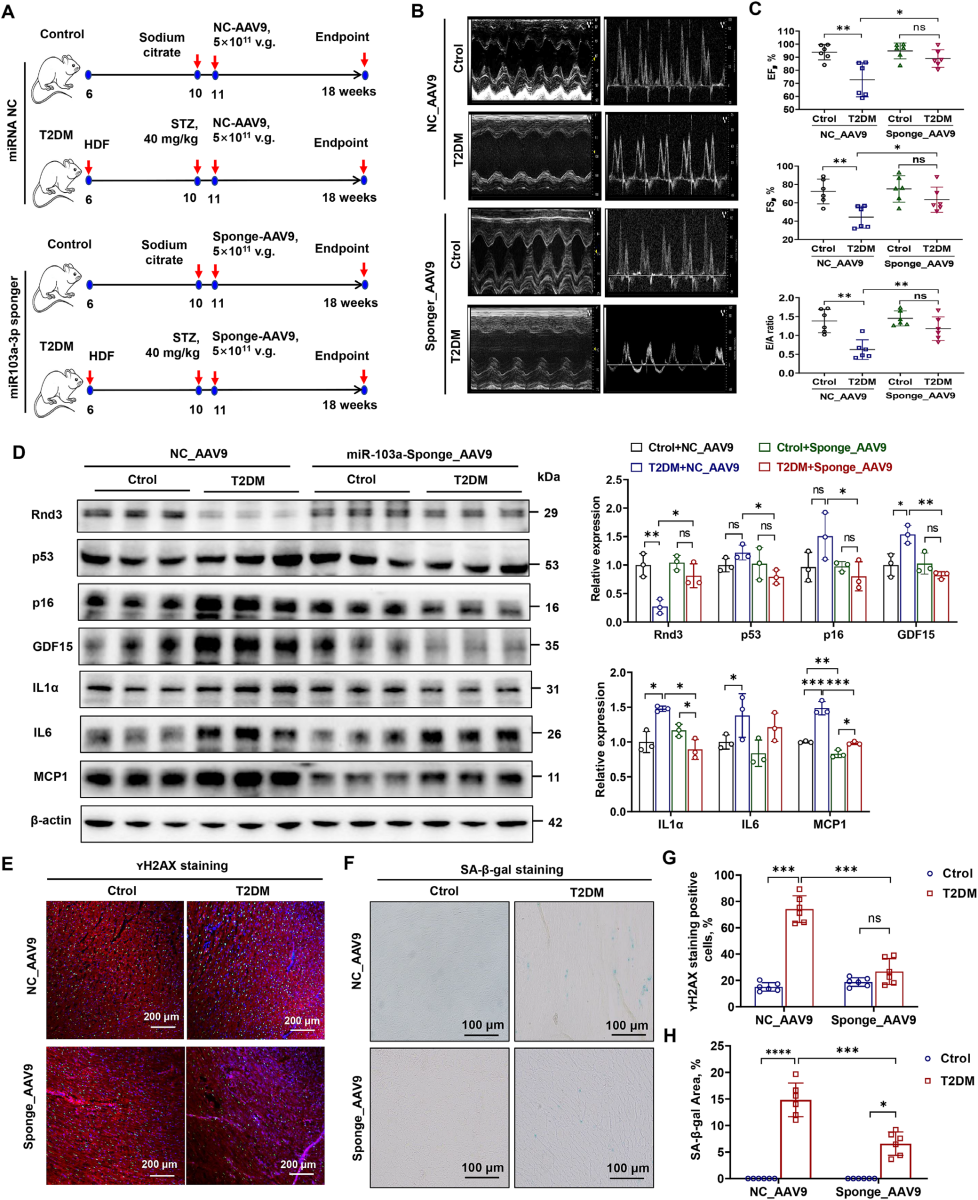
Cardiac function and cellular senescence analysis in rats treated with miR‐103a‐3p sponges. (A) Schematic diagram of the establishment of T2DM rats. (B) Representative images of echocardiography. (C) Cardiac function in rats with or without miR‐103a‐3p inhibition. Data were analyzed using an unpaired *t*‐test; *n* = 6, *p < 0.05, **p < 0.01. ns, no significance. (D) Western blot analysis of Rnd3, cell cycle‐related proteins p53 and p16, and SASP‐related markers MCP1, IL‐1α, IL‐6, and GDF15 in rats with or without miR‐103a‐3p inhibition. β‐actin served as the internal reference. Data were analyzed using an unpaired *t*‐test; *n* = 3, *p < 0.05, **p < 0.01. ns, no significance. (E, G) Immunofluorescence staining and quantitative analysis of γH2AX in heart tissue from rats with or without miR‐103a‐3p inhibition. Data were analyzed using an unpaired *t*‐test; *n* = 6, ***p < 0.001. ns, no significance. (F, H) SA‐β‐galactosidase staining in frozen heart sections of rats with or without miR‐103a‐3p inhibition. Data were analyzed using an unpaired *t*‐test; *n* = 6, *p < 0.01, ***p < 0.001, ****p < 0.0001.

To evaluate the in vivo effects of miR‐103a‐3p inhibition, we constructed a T2DM rat model using a high‐fat diet and STZ injection. 1 week later, rats received tail vein injections of AAV9 vectors overexpressing miR‐103a‐3p sponges or a negative control (Figure 7A, Table S7). miR‐103a‐3p sponge treatment significantly improved EF, FS, and E/A ratios compared to controls (Figure 7B,C). Western blotting confirmed that sponge treatment restored Rnd3 protein levels and reduced senescence marker expression (p53, p16, GDF15, IL‐1α, and MCP1) (Figure 7D), consistent with the qPCR results (Figure S8). SA‐β‐gal and γH2AX staining further demonstrated reduced senescence in diabetic myocardial cells (Figure 7E–H). These results suggest that inhibiting miR‐103a‐3p to restore *Rnd3* expression is an effective strategy for combating diabetes‐induced myocardial senescence.

### 2.7 Downregulation of *Rnd3* Induces a Cellular Senescence Phenotype in Cardiomyocytes by Activating the STAT3 Pathway

**FIGURE 8:**
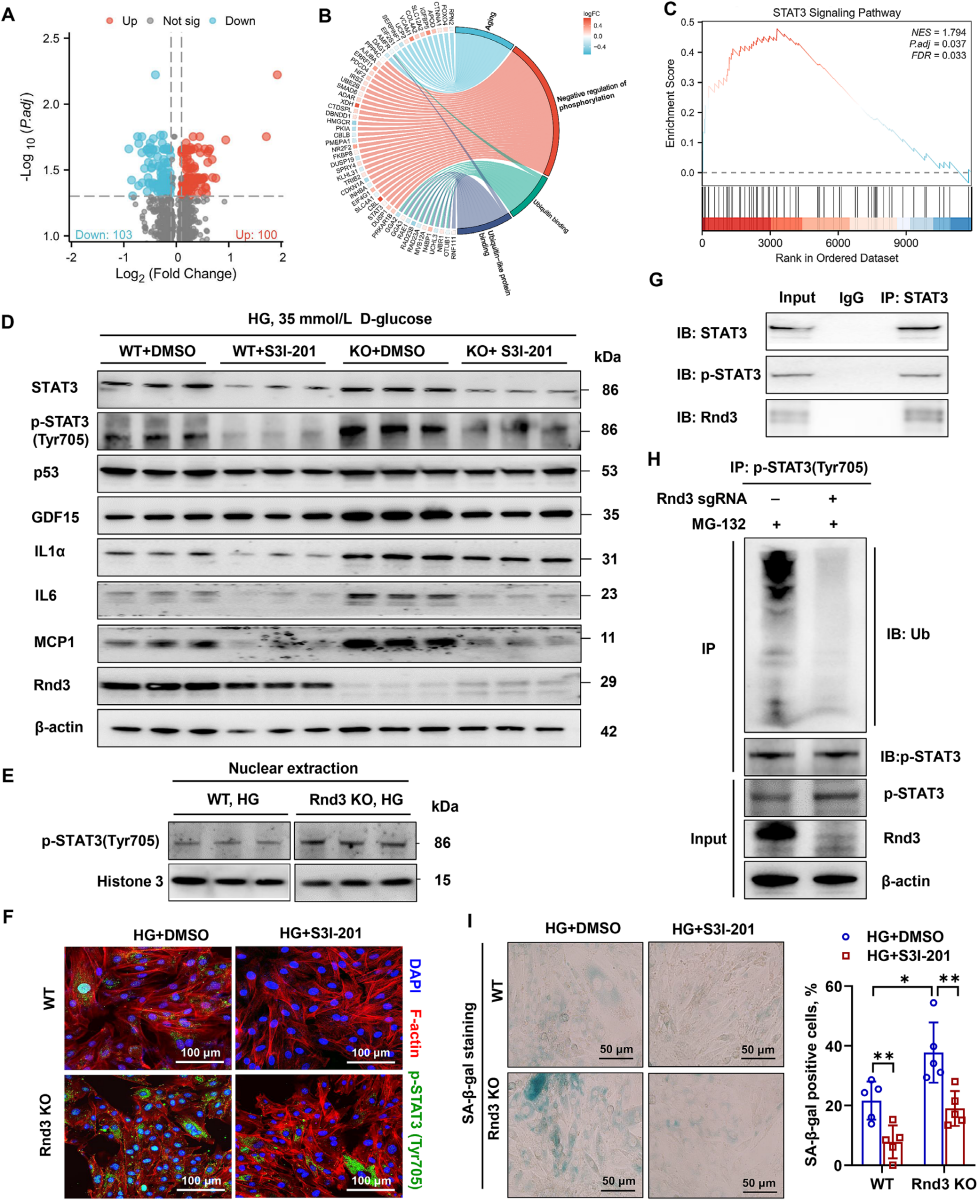
Interaction of Rnd3 with STAT3 in cardiomyocytes and its role in high glucose (HG)‐induced cellular senescence. (A) Volcano plot showing differentially expressed genes (DEGs) in *Rnd3* knockout or wild‐type H9C2 cells, with the threshold set as fold change ≥ 1.5 and *p* < 0.05. (B) Circos plot showing DEGs related to aging, phosphorylation, and ubiquitination after *Rnd3* knockout, based on GO analysis. (C) Gene Set Enrichment analysis showing positive regulation of STAT3 signaling in *Rnd3* knockout H9C2 cells. (D) Western blot analysis of STAT3 activation and cellular senescence in HG (35 mmol/L D‐glucose)‐treated H9C2 cells following treatment with the STAT3 inhibitor S3I‐201. *n* = 3, β‐actin served as the internal reference. (E) Upregulation of nuclear p‐STAT3 (Tyr705) in H9C2 cells following 48‐h HG (35 mmol/L D‐glucose) stimulation. *n* = 3, Histone 3 served as the internal reference. (F) Immunofluorescence detection of p‐STAT3 (Tyr705) expression and intracellular localization in HG (35 mmol/L D‐glucose)‐treated H9C2 cells following S3I‐201 treatment. (G) Interaction between Rnd3, STAT3 and p‐STAT3 assessed by co‐immunoprecipitation. (H) Ubiquitination‐mediated degradation of p‐STAT3 (Tyr705) in wild‐type and *Rnd3* knockout H9C2 cardiomyocytes. (I) STAT3 inhibitor S3I‐201 suppresses HG (35 mmol/L D‐glucose)‐induced H9C2 cardiomyocyte senescence. *n* = 5, unpaired *t*‐test, *p < 0.05, **p < 0.01.

To elucidate the mechanisms underlying *Rnd3* downregulation‐induced cardiac senescence, we reanalyzed our gene chip data from H9C2 cells with or without *Rnd3* knockout (Shao et al. 2020). Differential gene expression and Gene Ontology (GO) clustering revealed *Rnd3*'s involvement in aging, phosphorylation, and ubiquitination (Figure 8A,B). Gene set enrichment analysis (GSEA) indicated that *Rnd3* significantly regulates the STAT3 pathway (Figure 8C). In T2DM rats, phosphorylated STAT3 (p‐STAT3, Tyr705) levels were elevated, an effect mitigated by *Rnd3* overexpression and exacerbated by *Rnd3* knockout (Figure S9a,b). HG‐stimulated p‐STAT3 (Tyr705) accumulation was similarly modulated in vitro (Figure S9c,d). STAT3 inhibition with S3I‐201 reduced p‐STAT3(Tyr705) levels and SASP‐related proteins but not p53 or *Rnd3* expression (Figure 8D, Figure S9e). Notably, HG stimulation markedly activated nuclear accumulation of p‐STAT3 (Tyr705) in *Rnd3* knockout cardiomyocytes (Figure 8E), while S3I‐201 attenuated this effect (Figure 8F). Mechanistically, Rnd3 was found to bind to total STAT3 in cardiomyocytes (Figure 8G). Additionally, *Rnd3* knockout led to obviously attenuated p‐STAT3(Tyr705) rather than total STAT3 ubiquitination through the proteasome (Figure 8H, Figure S10). Finally, inhibition of p‐STAT3(Tyr705) with S3I‐201 reduced the percentage of SA‐β‐gal‐positive cells in HG‐induced *Rnd3* knockout H9C2 cells (Figure 8I). These findings highlight STAT3's role in *Rnd3* downregulation‐induced cardiomyocyte senescence under diabetic conditions.

## 3 Discussion

Rnd3 has garnered worldwide attention due to its association with cancers (Jie et al. 2015). Our prior studies examined the relationship between Rnd3 and cardiovascular diseases (Dai et al. 2019; Jie et al. 2015; Yue et al. 2016, 2014). We proposed that Rnd3 plays a crucial role in vascular and cardiac injury (Wu et al. 2021; Zhang et al. 2022, 2023; Hu et al. 2024; Ge et al. 2025), suggesting its potential therapeutic value in cardiovascular disease. Here, we explored our understanding of Rnd3 function by demonstrating its role in diabetes‐induced cardiomyocyte senescence. Our findings demonstrated that diabetes‐induced upregulation of miR‐103a‐3p suppressed *Rnd3* expression, activated STAT3 signaling, and promoted cardiomyocyte senescence. Targeted inhibition of miR‐103a‐3p or overexpression of *Rnd3* successfully restored Rnd3 expression in DCM and reversed cardiomyocyte senescence. These results offer a promising therapeutic strategy for the prevention and treatment of DCM‐associated senescence.

According to the International Diabetes Federation, the global diabetic population was approximately 463 million in 2019 and is expected to rise to 578 million by 2030 (Saeedi et al. 2019). The global rise in diabetes prevalence has drawn significant attention to its cardiovascular complications. Using the GEO database, we identified downregulated *Rnd3* mRNA levels in diabetic patients, a finding later validated in our clinical cohort. In our T2DM cohort, *Rnd3* mRNA levels correlated negatively with age, and *Rnd3* levels were associated with myocardial injury markers and cardiac function. Experiments on aged rats further supported these findings. These findings supported a connection among *Rnd3*, aging, and myocardial injury.

To clarify further the relationship between *Rnd3* and cardiomyocyte senescence in diabetes, we generated *Rnd3* knockout H9C2 cells and primary neonatal cardiomyocytes and found that HG‐stimulated knockout cells exhibited pronounced senescence, including cell cycle arrest and SASP expression, as SASP is known to play a role in regulating cellular senescence (Birch and Gil 2020). While *Rnd3* knockout amplified T2DM‐induced myocardial damage, *Rnd3* overexpression restored heart function and alleviated SASP in T2DM rats. Given the clinical relevance of cellular senescence in cardiac disease management (Mehdizadeh et al. 2022), targeting *Rnd3* to reverse cardiomyocyte senescence could be a significant advance in DCM treatment. Although the relationship between *Rnd3* and cardiovascular disease is known, this is the first report to connect *Rnd3* to DCM cardiomyocyte senescence.

To elucidate the mechanism by which diabetes inhibits *Rnd3* expression, we focused on the epigenetic modification mechanisms of miRNAs, rather than classical signaling pathways. miRNAs, which are pivotal regulators of gene silencing (Ha and Kim 2014), have garnered increasing recognition as potential therapeutic targets for cardiovascular senescence (Jusic et al. 2022; Li et al. 2020). Through miRNA sequencing, we identified miR‐103a‐3p as a conserved miRNA upregulated by HG stimulation, which directly targets the *Rnd3* 3′UTR. We then conducted bioinformatics analysis to identify the downstream target genes of miR‐103a‐3p, initially revealing its involvement in cellular senescence and cardiovascular diseases. Using cardiomyocytes cultured under HG conditions, heart tissues from T2DM rats, and plasma samples from T2DM individuals, we consistently observed a significant elevation of miR‐103a‐3p expression. Application of a specific antagonist for miR‐103a‐3p markedly downregulated the miR‐103a‐3p levels in hearts from diabetic rats and in HG‐treated cardiomyocytes. Such downregulation concurrently attenuated the degree of cardiomyocyte senescence and damage. These findings confirm the potential value of miR‐103a‐3p in DCM treatment. miR‐103a‐3p has been implicated in several human diseases such as Parkinson's disease (Zhou et al. 2020), cancers (Sun et al. 2020; Xu et al. 2022), and nephropathy (Lu et al. 2019). Within the cardiovascular system, Zhang et al. (2019) proposed that miR‐103a‐3p is associated with hypoxia and reoxygenation (H/R)‐induced cardiomyocyte autophagy and apoptosis. Our study not only elucidates the molecular mechanism by which diabetes inhibits *Rnd3* expression via miR‐103a‐3p upregulation, but also provides a robust foundation for the clinical translation of miR‐103a‐3p‐targeted interventions to treat DCM. As mentioned in a meta‐analysis, miR‐103a‐3p may serve as a biomarker for T2DM (Zhu and Leung 2023). The results from our research support this conclusion.

Rnd3 mitigates oxidative stress, fibrosis, and inflammation by inhibiting ROCK1, nuclear factor kappa‐B, Notch, and TGF‐β signaling pathways, which are also critical drivers of cardiac senescence (Dai et al. 2019; Lin et al. 2013; Zhang et al. 2022). Our prior research showed that *Rnd3* knockout activated the Jak/STAT pathway (Shao et al. 2020). STAT3 signaling is pivotal for cell‐to‐cell communication in the heart (Haghikia et al. 2014). Consistent with previous reports, our study demonstrated that HG stimulation and *Rnd3* knockout upregulated p‐STAT3 and SASP‐related inflammatory factors (Sun et al. 2021; Yang et al. 2022). Notably, we identified for the first time that Rnd3 interacted with STAT3 in cardiomyocytes, inducing ubiquitination‐mediated degradation of p‐STAT3 and attenuating its nuclear translocation. This mechanism highlights the role of STAT3 in diabetes‐induced cardiomyocyte senescence, driven by reduced *Rnd3* expression.

STAT3 signaling has diverse roles in the cardiovascular system, functioning protectively or pathogenically under different conditions. For example, IL‐6‐mediated STAT3 activation mediated ventricular hypertrophy and fibrosis (Kumar et al. 2019; Zhao et al. 2016). Conversely, granulocyte colony‐stimulating factor prevented post‐infarction remodeling via STAT3 activation (Harada et al. 2005). Moreover, cardiac‐specific knockout of STAT3 exacerbated subacute ventricular remodeling in mice with myocardial infarction (Enomoto et al. 2015). In our study, *Rnd3* knockout elevated SASP‐related factors, such as IL‐6, MCP1, and IL‐1α, by activating p‐STAT3. STAT3 phosphorylation and SASP factor expression are mutually reinforcing processes (Yan et al. 2013; Yasuda et al. 2021; Zhao et al. 2016). To further clarify the upstream and downstream relationship between Rnd3, SASP, and STAT3, we used the highly selective STAT3 inhibitor S3I‐201 (Siddiquee et al. 2007). Interestingly, regardless of the group (wild type or Rnd3 knockout), treatment of H9C2 cells with S3I‐201 significantly inhibited the expression of SASP‐related factors, such as IL‐1α, IL‐6, and MCP1, but not Rnd3, and was accompanied by a reduction in SA‐β‐gal‐positive cells. These findings suggest that the activation of STAT3 is a key pathway involved in diabetes‐/HG‐induced cardiomyocyte senescence through inhibiting Rnd3. A recent report highlighted that blocking age‐related inflammatory responses was crucial in treating aging and neurodegeneration (Gulen et al. 2023). Our study showed that targeted intervention of miR‐103a‐3p/Rnd3/STAT3 signaling in diabetes blocked the expression of SASP‐related inflammatory factors and thus reduced cardiomyocyte senescence, consistent with the aforementioned findings.

This study has some limitations. First, the heart consists of various cell types, but we focused solely on the effect of Rnd3 expression defects on cardiomyocyte senescence. Future studies should explore the role of other cell types in cardiac senescence. Second, Rnd3 dysregulation likely affects multiple signaling pathways. Advanced multiomics techniques, such as proteomics, could identify additional pathways influenced by Rnd3.

In conclusion, our study demonstrated that diabetes induced cardiomyocyte senescence through upregulation of miR‐103a‐3p, which suppressed *Rnd3* expression and activated the STAT3 signaling, thereby promoting SASP‐related factor expression. Strategies targeting the miR‐103a‐3p/Rnd3/STAT3 axis, such as *Rnd3* overexpression, miR‐103a‐3p inhibition, or pharmacological STAT3 blockade, effectively counteracted cardiomyocyte senescence and preserved cardiac function in diabetic models. These findings offer a promising framework for developing novel DCM therapies.

## 4 Materials and Methods

### 4.1 Bioinformatics Analysis

Normalized probe expression matrices for diabetes were obtained from GEO (https://www.ncbi.nlm.nih.gov/gds/) under accession numbers GSE23561, GSE95849, and GSE13760. Probe IDs were converted into gene symbols, and batch effects across datasets were removed using the removeBatchEffect function in the limma package (v3.52.4). *Rnd3* expression levels across cardiac tissue cell types were analyzed using the Human Protein Atlas (https://www.proteinatlas.org/). Annotated sequencing data identified cell types using tissue and cell‐specific markers, with primary cell types selected for comparative analysis of *Rnd3* expression. Differentially expressed genes (DEGs) from *Rnd3* knockout H9C2 cells were reanalyzed using previous data (Shao et al. 2020), with thresholds set at fold change (FC) ≥ 1.5 and *p* < 0.05. Results were visualized using the ggplot2 package (v3.4.4). GSEA was performed with the clusterProfiler package (v4.4.4), and Z‐scores for enriched terms were calculated using the GOplot package (v1.0.2) based on logFC values.

### 4.2 Animal Models

Five‐week‐old male wild‐type SD rats were purchased from Slaccas Company (Changsha, China). T1DM and T2DM models were established by STZ injection. Age‐related senescence models were created by raising SD rats to 96 weeks of age. Cardiomyocyte‐specific *Rnd3* knockout rats were generated by crossing Flop^+/+^ rats with α‐Myosin heavy chain (αMHC^Cre+/+^) rats (Cyagen, Suzhou, China). For T1DM, rats were intraperitoneally injected with 65 mg/kg STZ (#60256ES80; Yeasen, Shanghai, China). For T2DM, rats were fed a high‐fat diet (#PSY1102; Slaccas) for 1 month, followed by a 40 mg/kg STZ injection. Control rats received a normal diet and equivalent citrate buffer injection. Blood glucose concentrations were monitored, with FBG ≥ 11 mmol/L or random blood glucose (RBG) ≥ 16.7 mmol/L indicating successful model establishment. 1 week later, animals received either control AAV9 (10^12^ v.g/rat; Genechem, Shanghai, China), cTnI‐*Rnd3*‐AAV9 (10^12^ v.g/rat; Genechem), or miR‐103a‐3p sponges‐AAV9 (5 × 10^11^ v.g/rat; Genechem). Cardiac function was assessed by ultrasound, and left ventricular tissues were collected for further analysis. All experiments adhered to the ethical requirements of Hainan Medical University (Approval number: KYLL‐2020‐048).

### 4.3 Human Blood Samples and Clinical Data

Between March 1 and November 1, 2023, patients from the Cardiology Department, First Affiliated Hospital of Hainan Medical University, were recruited based on predefined inclusion and exclusion criteria. A total of 74 patients were enrolled, with serum and blood cells collected for RNA extraction. Clinical data for each patient were recorded. The study protocol was approved by the Ethics Committee of the First Affiliated Hospital of Hainan Medical University (Approval number: 2023‐KYL‐096).

### 4.4 Elisa

Peripheral blood mononuclear cells were obtained by lysing whole blood samples with blood cell lysis buffer (#C3702; Beyotime, Shanghai, China) and centrifugation. Total proteins were extracted using RIPA buffer (#P0013B; Beyotime), and concentrations were measured via the BCA assay. Rnd3 concentrations were measured using a human Rnd3 ELISA kit (#MM‐64412H1; Meimian, Jiangsu, China) following the manufacturer's protocol, with 0.2 mg of protein used per sample.

### 4.5 Cell Culture and Treatment

Primary neonatal rat cardiomyocytes (#AW‐YCR156) were sourced from Abiowell Biotechnology Co. Ltd. H9C2 cardiomyocytes were obtained from the Shanghai Stem Cell Bank, and AC16 cardiomyocytes were procured from ATCC (Manassas, VA, USA). Cells were cultured in DMEM supplemented with 10% fetal bovine serum and maintained at 37°C with 5% CO_2_. To study the effects of glucose, cells were treated with DMEM containing glucose concentrations of 5.5 mmol/L (NG), 12.5 mmol/L, 35 mmol/L, or 50 mmol/L (HG). HG conditions were maintained for 72 h to establish senescence models. To investigate the effect of miR‐103a‐3p, H9C2 and AC16 cells were treated with 5.5 mmol/L D‐glucose and 35 mmol/L D‐glucose plus or with or without an miR‐103a‐3p inhibitor (10 μmol/L; Ruibo, Guangzhou, China) for 72 h. To examine the role of STAT3 signaling in HG‐stimulated cardiomyocytes, the STAT3 inhibitor S3I‐201 (10 μmol/L; #HY‐15146, MCE China, Shanghai) was used to maintain the 24‐h HG‐pretreated cells for an additional 48 h.

### 4.6 *Rnd3* Knockout in H9C2 Sells

*Rnd3* knockout in H9C2 cells was performed using lentiviral vectors as previously described (Shao et al. 2020). sgRNA (AGCAGTCCTTTGCGAAGACG) was cloned into the GV392 vector containing Cas9 recombinase (#00595–1, Genechem). Lentiviral particles infected H9C2 cells at a multiplicity of infection of 100. Control cells were transfected with empty vectors. After 72 h, puromycin (2 μg/mL) was applied for 7 days to select transfected cells. Knockout efficiency was confirmed via quantitative PCR and western blotting.

### 4.7 SA‐β‐Galactosidase Staining

A SA‐β‐galactosidase staining kit (#C0602; Beyotime) was used to identify senescent cardiomyocytes. Cells cultured in six‐well plates were retrieved from the incubator, and the culture media were aspirated. Cells were washed twice with pre‐cooled phosphate‐buffered saline (PBS). Frozen heart tissue sections were thawed and immersed in PBS three times, each for 5 min. Next, 1 mL of SA‐β‐gal staining fixative was added to each well or section and incubated at room temperature for 15 min. The fixative was aspirated, and wells were washed three times with 1 mL of pre‐chilled PBS, each for 3 min. After removing PBS, 1 mL of staining working solution was prepared and added to each well. The solution consisted of SA‐β‐gal staining solutions A:B:C in a 1:1:93:5 ratio. The six‐well plates were sealed with cling film and incubated overnight at 37°C. The following day, stained cells were photographed under a conventional optical microscope. For cultured cells, the percentage of SA‐β‐gal‐positive cells was analyzed using ImageJ software (http://imagej.nih.gov/ij/). For tissue sections, the area of SA‐β‐gal‐positive cells was measured with ImageJ.

### 4.8 Immunofluorescence Staining

Adherent cells and frozen cardiac tissue sections were stained with a DNA damage detection kit (#C2035S; Beyotime). For cells in confocal dishes, the medium was aspirated, followed by two PBS washes. Frozen sections were immersed in PBS for 1 min. Each dish or section received 600 μL of fixative and was incubated at room temperature for 15 min, then washed six times with PBS, 1 min each. A blocking solution (200 μL) was added to each sample and incubated for 20 min at room temperature. Post‐blocking, 200 μL of γH2AX or p‐STAT3 rabbit monoclonal antibody was added and incubated overnight at 4°C. Samples were then washed six times with PBS, 3 min each, before adding a fluorescent secondary antibody and incubating for 1 h at room temperature. Following this, dishes or sections were washed six times with PBS, 3 min each. A nuclear stain‐containing mounting medium was applied, and images were captured using a confocal microscope (FV3000; Olympus, Japan). The percentage of γH2AX‐positive cells was quantified using ImageJ.

### 4.9 Immunoblotting and Co‐IP Assays

Total protein was extracted from cells or heart tissue using RIPA buffer (#P0013B; Beyotime). For co‐immunoprecipitation (Co‐IP), cells were lysed with IP lysis buffer, incubated overnight at 4°C with corresponding antibodies, and processed using the Co‐IP kit (#C2035S; Beyotime) as per the manufacturer's instructions. Nuclear proteins were isolated using the NE‐PER Kit (#78833; Thermo Fisher, Massachusetts, USA). Protein concentrations were determined using the bicinchoninic acid method, and 20–40 μg of protein was loaded onto SDS‐PAGE gels for separation. Proteins were then transferred to PVDF membranes. Membranes were washed three times with TBST for 3–5 min each on a shaker and blocked at room temperature for 1 h using QuickBlock Solution (#P0252; Beyotime). The following primary antibodies were used: Anti‐STAT3 antibody (1:2000; #PTM‐5090, PTM BIO, Hangzhou, China); Anti‐Phospho‐STAT3 (Tyr705) antibody (1:2000; #9145, CST, MA, USA); Anti‐Rnd3 antibody (1:2000; #DF12311, Affinity, Jiangsu, China); Anti‐MCP1 antibody (1:1000; #ab214819, Abcam, United Kingdom); Anti‐IL‐6 antibody (1:1000; #ab259341, Abcam); Anti‐IL‐1α antibody (1:2000; #DF6893, Affinity); Anti‐GDF15 antibody (1:2000; #ab206414, Abcam); Anti‐p53 antibody (1:2000; #PTM‐6319, PTM BIO); Anti‐p16 antibody (1:2000; #ab51243, Abcam); Anti‐β‐actin antibody (1:1000; #PTM‐5706, PTM BIO); and VeriBlot for IP Detection (1:1000; #ab131366, Abcam). The next day, membranes were washed three times with TBST for 3 min each, followed by incubation with secondary antibodies at room temperature for 1 h. After washing five times with TBST for 3 min each, enhanced chemiluminescence reagents were applied, and images were captured using an automatic imaging system (Tanon‐ABLX5, Shanghai). Grayscale values of protein bands were analyzed using ImageJ software.

### 4.10 Quantitative Real‐Time PCR

Total RNA was extracted from cells, heart tissue, and serum using the Eastep Super Reagent Kit (#LS1040; Promega, USA). The Hifair III 1st Strand cDNA Synthesis SuperMix for quantitative reverse transcription‐polymerase chain reaction (RT‐PCR) (gDNA digester plus) (#11141ES60; Yeasen, Shanghai, China) was used for the reverse transcription of cDNA, while the Hifair miRNA 1st Strand cDNA Synthesis Kit (Add A method) (#11148ES10; Yeasen) was used for the reverse transcription of miRNA. The Hieff qPCR SYBR Green Master Mix (Low Rox Plus) (#11202ES08; Yeasen) was used for PCR. β‐actin was used as the internal mRNA reference. U6 was used as the internal control for miRNA expression in both tissue and cell samples. To ensure reliable miRNA qPCR detection in serum, miR‐23a was selected as the internal control due to its relative stability in serum, remaining unaffected by diabetes and hemolysis.

PCR primers were designed and synthesized by Sangon Biotech (Shanghai, China) and are listed in Table S8.

### 4.11 MicroRNA Sequencing

MicroRNA sequencing was conducted using the Beijing Genomics Institute platform (Shenzhen, China) (Wang et al. 2016). AC16 cells were cultured in normal glucose (NG) and high glucose (HG) media for 48 h. Total RNA was extracted using the MiniBEST Universal RNA Extraction Kit (#9767; Takara, Japan). RNA samples underwent electrophoresis on polyacrylamide gels, and small RNAs (18–30 nt) were recovered from the gels. Subsequent library preparation included linking the 3′ and 5′ ends, reverse transcription, and PCR amplification. Amplified products were electrophoresed again, and target bands were extracted from gels and preserved in Ethidium bromide buffer. Libraries underwent quality control according to the product requirements. PCR products were denatured into single strands, circularized to construct single‐strand circular DNA, and digested to remove linear DNA. Rolling circle replication was then performed to create DNA nanoballs, which were loaded onto high‐density DNA nanochips for sequencing using combined probe anchoring polymerization technology. Raw sequencing data were filtered, and clean reads were aligned to the reference genome using Bowtie2 (v2.2.5) to predict miRNA. Reads were annotated sequentially in the following order: miRbase > pirnabank > snoRNA > Rfam > other sRNAs, ensuring each small RNA (sRNA) was assigned a single annotation. Novel miRNAs were predicted using the Dr. Tom system and miRDeep2 software. Differential gene expression was analyzed with DEGseq, using thresholds of *Q*‐value ≤ 0.05 or false discovery rate ≤ 0.001. Target genes of differentially expressed miRNAs were predicted using RNAhybrid, miRanda, and TargetScan software. Only target genes identified by all three databases were selected. GO and KEGG enrichment analyses were performed on these genes using Phyper. Significantly enriched genes were identified with Bonferroni correction (corrected *p* ≤ 0.05).

### 4.12 Dual Luciferase Experiment

Firefly/Renilla luciferase assays were performed to evaluate *Rnd3* 3′UTR promoter activity. The R*nd3* 3′UTR and its mutated version were cloned into the GV272 plasmid (SV40‐firefly_Luciferase‐MCS). To mutate the miR‐103a‐3p binding site, TGCTGC in the rat *Rnd3* 3′UTR was replaced with GTAGTA. The miR‐103a‐3p precursor, miR‐103a‐3p‐1, was cloned into the GV268 plasmid (CMV‐MCS‐SV40‐Neomycin). 293 T cells were seeded in 24‐well plates and co‐transfected with 0.1 μg of *Rnd3* 3′UTR plasmid, 0.4 μg of miR‐103a‐3p‐1, and 0.4 μg of negative control (NC) using X‐tremegene HP reagents (#06366236001, Roche, Switzerland). Positive control cells were transfected with 0.4 μg of hsa‐mir‐146b and 0.1 μg of TRAF6 3′UTR. All cells were cultured in DMEM medium containing 10% FBS. 48 h post‐transfection, firefly and renilla luciferase activity was measured using the Dual‐Luciferase Reporter Assay System (#E1910, Promega, USA) according to the manufacturer's instructions. Luciferase activity was expressed as the ratio of firefly to renilla luminescence. All plasmids were constructed by Shanghai Genechem Company.

### 4.13 Echocardiography

Transthoracic echocardiography was performed in all experimental animals using a VisualSonics VeVo 2100LT system (FUJIFILM VisualSonics, Toronto, Canada), as previously described (Wu et al. 2024). The animals were continuously anesthetized with 3% isoflurane. Under B‐mode ultrasound, the probe was placed on the left side of the sternum and adjusted along the long axis of the heart to obtain a clear long‐axis echocardiogram. M‐mode echocardiography was then used to collect videos of cardiac motion. Subsequently, Doppler ultrasound was used to monitor blood flow at the mitral valve to measure the E/A ratio. The results of the echocardiogram were measured by VEVO LAB (v5.5.1).

### 4.14 Statistical Analysis

GraphPad Prism 9 (GraphPad Software, USA) was used for statistical analysis. Data normality was assessed via the Shapiro–Wilk test and QQ plots. Parametric data are presented as mean ± SD and analyzed using t‐tests or ANOVA with Bonferroni post hoc tests. Nonparametric data are shown as median ± interquartile range and analyzed using the Mann–Whitney test. Spearman and Pearson correlation analyses were applied to clinical data. *p* < 0.05 was considered statistically significant.

## Author Contributions

L.W., X.Z., S.P., Y.C., C.L., Y.Z., J.X., K.S., J.Q., S.Z., and J.L. performed the experiments and collected the data. L.W. and W.J. prepared the original draft. X.L. collected the clinical samples and reviewed the data. L.W., X.Z., C.L., Y.W., and J.C. prepared the materials. Z.S. and X.L. searched the literature and performed statistical analyses. Z.S., J.G., and W.J. conceived and supervised the project. W.J. and J.G. acquired financial support. All of the authors read and approved the final manuscript.

## Conflicts of Interest

The authors declare no conflicts of interest.

## Supporting information


Data S1.

## Data Availability

The raw RNA‐seq data in our analyses have been uploaded to the Gene Expression Omnibus database (https://www.ncbi.nlm.nih.gov/geo) under accession numbers: GSE23561, GSE95849, GSE13760. Additional data, analytic methods, and study materials will be made available to researchers upon request.
