# Supplementary material for: Diabetes Advances Cardiomyocyte Senescence Through Interfering Rnd3 Expression and Function
Source: Aging Cell. 2025 Mar 2;24(6):e70031. doi: 10.1111/acel.70031 (PMC12151882; doi:10.1111/acel.70031)
Supplement: Supplementary file 1 — Data S1. [file ACEL-24-e70031-s001.pdf]

SUPPLEMENTAL MATERIALS

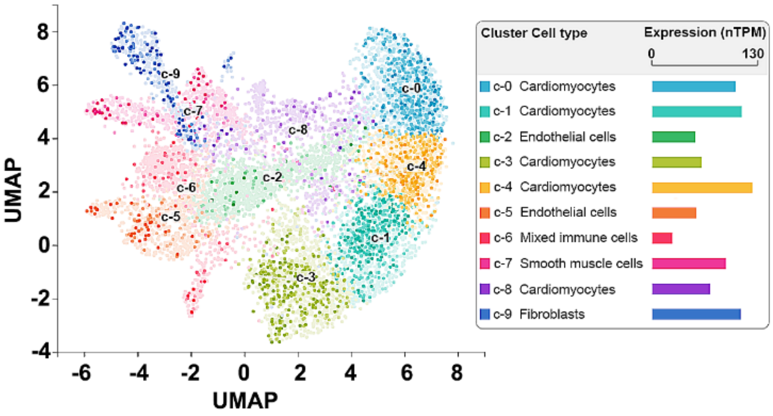

Figure S1. Expression of *Rnd3* mRNA in normal human heart tissue. Single-cell RNA sequencing shows *Rnd3* mRNA levels in normal human cardiac cell types based on the Human Protein Atlas database.

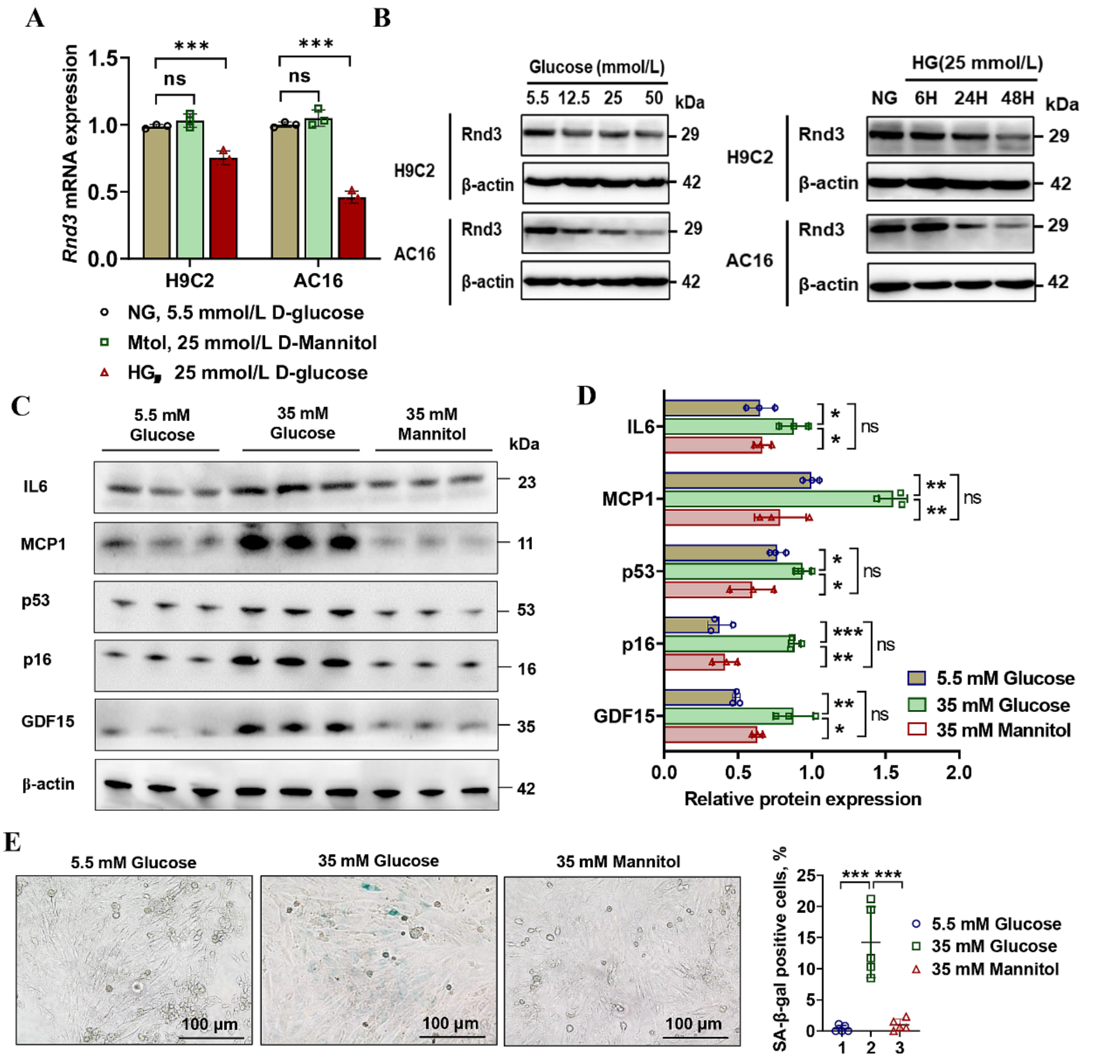

Figure S2. High glucose concentration inhibited the expression of Rnd and induced cardiomyocytes senescence independent of osmotic pressure. **(A)** H9C2 and AC16 cells were treated by normal

glucose (NG), D-mannitol (Mtol) and high glucose (HG) condition for 24 hours, then cells were harvested for RT-qPCR analysis of *Rnd3* mRNA. *n*=3, ****P*<0.001. ns, no significance. (**B**) H9C2 and AC16 cells were treated by NG and HG condition for different periods or concentrations, then cells were harvested for western blotting analysis of Rnd3 Protein. (**C**, **D**) The effect of osmotic pressure on cellular senescence related factor expression in H9C2 cells was detected by immunoblotting. Unpaired *t* test, ns, no significance, *n*=3, **P*<0.05, ***P*<0.01, ****P*<0.001. (**E**) SA-β-gal staining was used to evaluate the effect of osmotic pressure on senescence of H9C2 cells. *n*=5, ****P*<0.001.

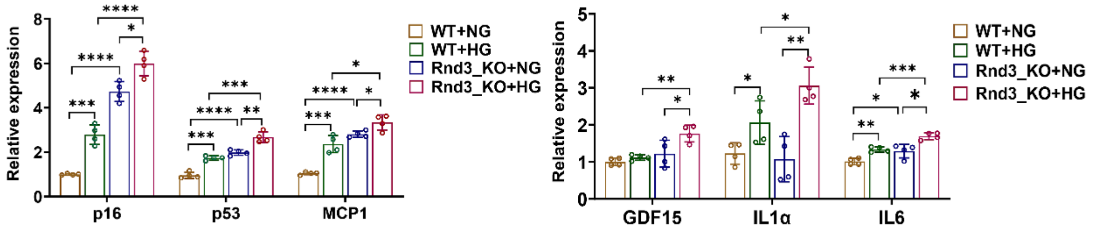

Figure S3. **Quantitative analysis charts of Western blot detection for aging related biomarkers**. Western blot detection of aging related biomarkers in NG (5.5 mmol/L D-glucose)-treated and HG (35 mmol/L D-glucose)-treated H9C2 cells. β-actin served as an internal reference. Data were analyzed by the unpaired t test, n=4, **P*<0.05, ***P*<0.01, ****P*<0.001, *****P*<0.0001.

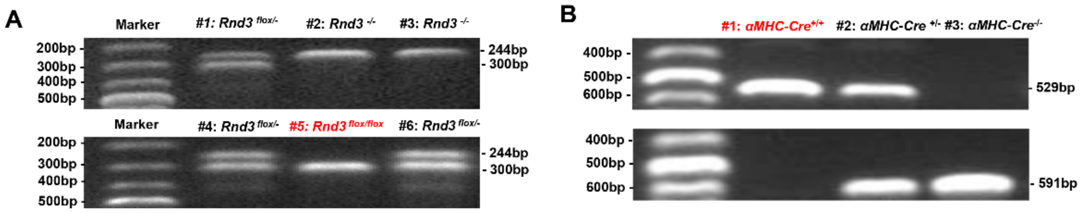

Figure S4. Genotyping of model rats. (A) PCR was performed using the following primers to identify the genotype of Flox rats. Among them, #5 (red) represents Flox$^{+/+}$ rats. Forward primer (F): 5'-GCA CCT ATG TAG AAG TCC AGG CTT G-3', Reverse primer (R): 5'-AAC TAA GAA GGA CCC TTT GAT CTA CC-3'. **(B)** PCR was performed using the following primers to identify the genotype of cardiomyocyte specific Cre expressing rats. Among them, #1 (red) represents Cre$^{+/+}$ rats. Forward primer(F): 5'- ATT CCT CCT TGA GTT GTG GCA CT-3', Reverse primer_1 (R1): 5'- TGG GCA TGT CTT CAA TCT ACC TC-3'. Reverse primer_2 (R2): 5'-ATG AAC AAA GGT TGG CTA TAA AGA G-3'. Finally, the cardiomyocyte specific *Rnd3* gene knockout rats were obtained by co-breeding # 5 rat in

(A) and # 1 rat in (B), and male offspring rats were used for related experiments.

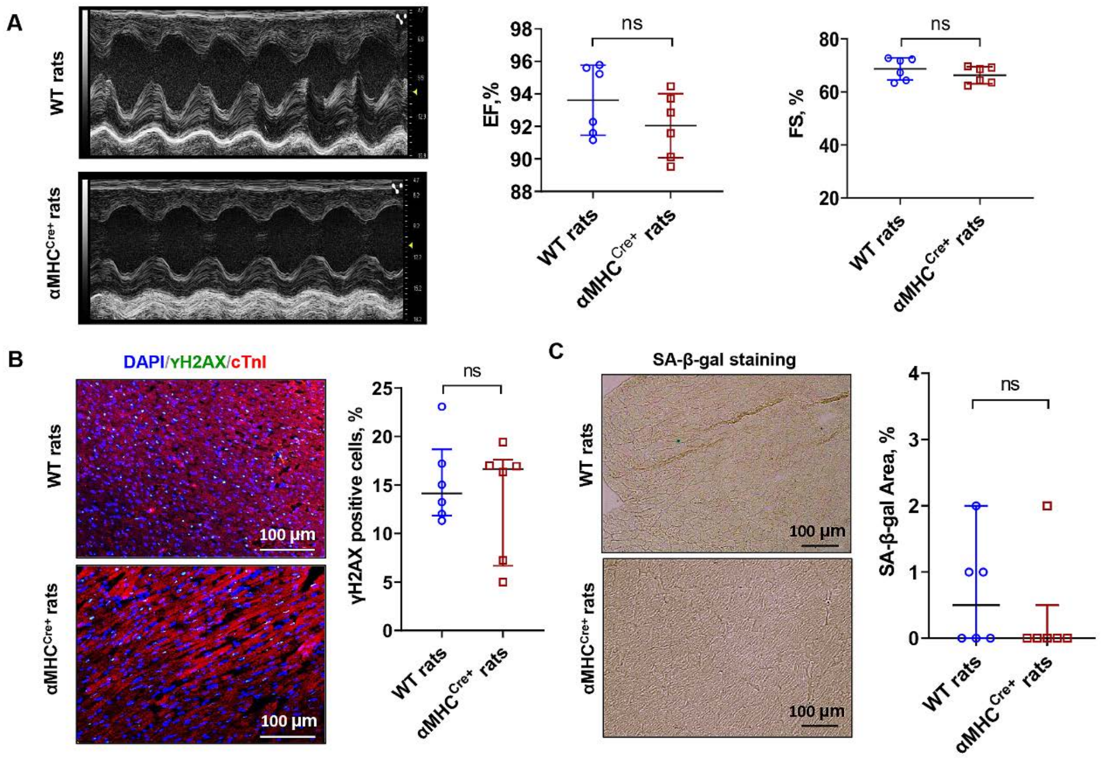

Figure S5. Comparison of cardiac function, myocardial aging, and DNA injury between αMHC^{Cre+} and wild type (WT) rats. (A) Cardiac function, Data were analyzed by the unpaired t test; n=5, ns, no significance. (B) Immunofluorescence staining of γH2AX in cardiac tissues. Data were analyzed by the unpaired t test; n=5, ns, no significance. (C) A-β-galactosidase staining in cardiac tissues. Data were analyzed by the unpaired t test; n=5, ns, no significance.

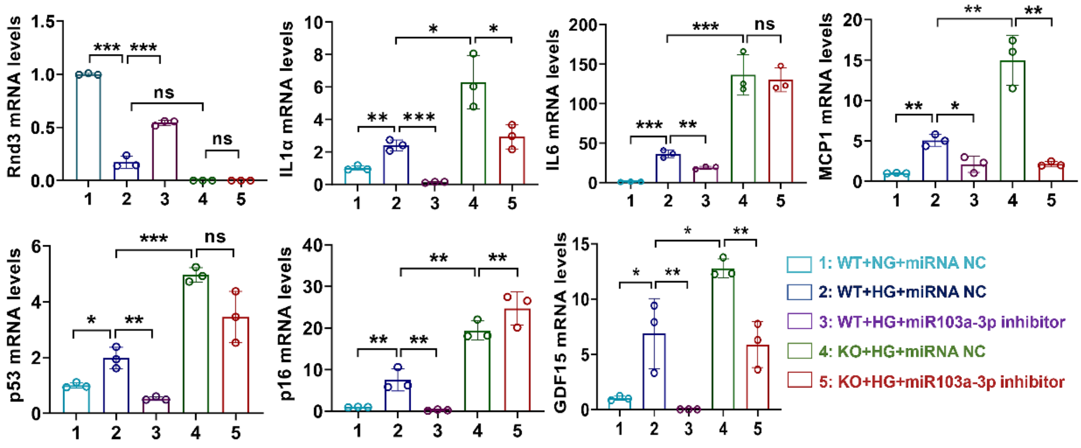

Figure S6. qPCR analysis of mRNA levels of cellular senescence markers in miR-103a-3p inhibitor treated H9C2 cells. *n*=3, Unpaired t test, **P*<0.05, ***P*<0.01, ****P*<0.001. ns, no significance.

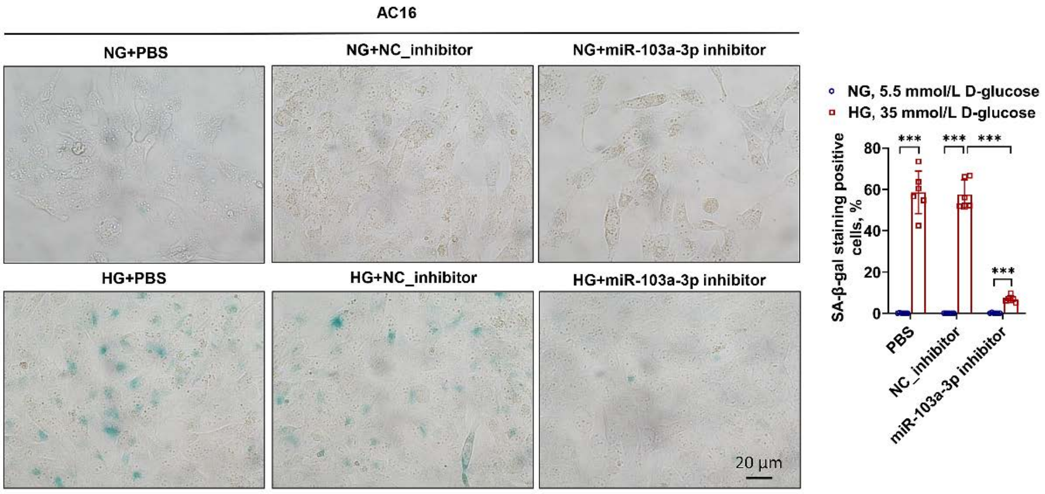

Figure S7. SA-β-galactosidase staining in miR103a-3p inhibitor (10 μmol/L) treated AC16 cells maintained in high glucose (HG, 35 mmol/L D-glucose) condition for 72 hours. AC16 cells maintained in normal glucose (NG, 5.5 mmol/L D-glucose) plus PBS buffer or negative control of miR103a-3p inhibitor **(**NC_inhibitor, 10 μmol/L**)** for 72 hours served as controls. Data were analyzed by the unpaired t test; *n*=5, ****P*<0.001.

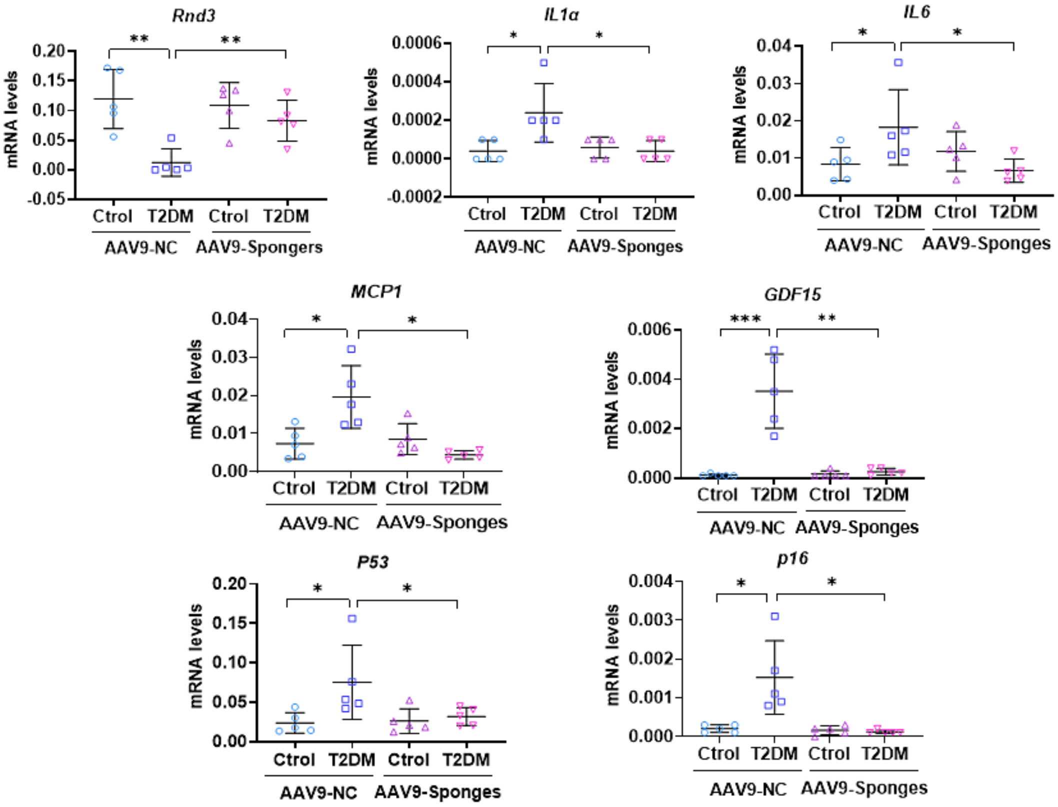

Figure S8. qPCR was used to analyze the expression of mRNA of *Rnd3* and cellular senescence markers. Diabetic and non-diabetic rats were injected with AAV9-miR-103a-3p sponges through the tail vein, and the control group was injected with the same titer of AAV9-NC virus. After 8 weeks, cardiac

tissues were used qPCR analysis of the expression of mRNA of *Rnd3* and cellular senescence markers. Data were processed by Livak method and expressed as mean ± standard deviation, *n*=5. **P*<0.05, ***P*<0.01, ****P*<0.001.

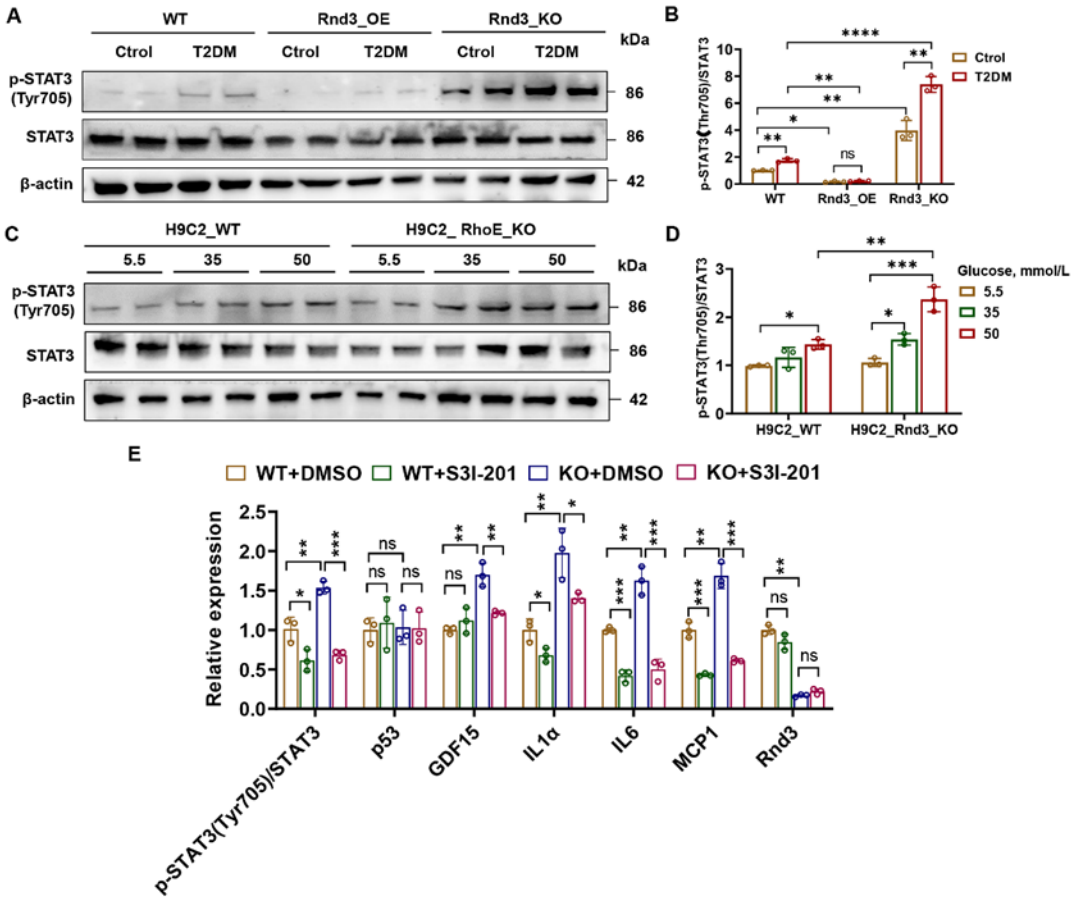

Figure S9. Effects of diabetes mellitus or HG on STAT3 phosphorylation in cardiac tissues and cardiomyocytes. (**A**, **B**) Western blot detection of p-STAT3 (Tyr705) and STAT3 protein levels in heart tissue of rats with or without *Rnd3* gene intervention. β-actin served as an internal reference. Data were analyzed by the unpaired t test; *n*=3, **P*<0.05, ***P*<0.01, *****P*<0.0001. ns, no significance. **(C, D)** Western blot detection of p-STAT3 (Tyr705) and STAT3 protein levels in HG-treated H9C2 cells. β-actin served as an internal reference. Data were analyzed by the unpaired *t* test; *n*=3, **P*<0.05, ***P*<0.01, ****P*<0.001. (**E)** Quantitative analysis of western blot detection of the STAT3 inhibitor S3I-201 on STAT3 activation and cellular senescence in HG-treated H9C2 cells. Data were analyzed by the unpaired *t* test; *n*=3, **P*<0.05, ***P*<0.01, ****P*<0.001, ns, no significance.

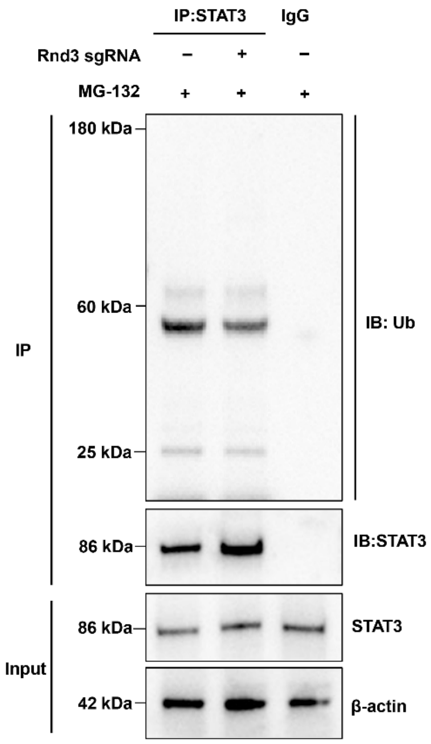

Figure S10. Ubiquitin-mediated total STAT3 degradation in wild and *Rnd3* knockout H9C2 cardiomyocytes. Cell lysis was incubated with anti-STAT3 antibodies then subjected to detection of Ub. The CO-IP results showed that the region between 60 and180 kDa did not showed any ubiquitin binding signs. Bands blow 60 kDa and near 25 kDa may refer to STAT3 antibodies reacted with HRP.

Table S1. Characteristics of clinical cohorts involved in *Rnd3* mRNA test

Variate	Overview (n=33)	Control (n=15)	Diabetes (n=18)	p-Value
Female, n(%)	11(33.33)	6(40.00)	5(27.78)	0.458
Man, n(%)	22(66.67)	9(60.00)	13(72.22)	
Rnd3, median[IQR]	0.001[0.000, 0.008]	0.004[0.001, 0.009]	0.000[0.000, 0.003]	0.019
Age, median[IQR]	59.79±10.53	56.87±10.51	62.22±9.90	0.155
FBG(mmol/l), median[IQR]	6.02[4.94, 9.62]	4.68[4.18, 5.29]	8.24[7.61, 11.87]	<0.001
BUN(mmol/l), median[IQR]	5.96[4.55, 7.60]	5.06[4.60, 6.71]	6.90[5.96, 7.30]	0.330
CREA(μmol/l), median[IQR]	73.90[61.60, 110.20]	70.40[65.50, 102.60]	78.80[62.70, 110.20]	0.868
BUN/CR, median[IQR]	0.07[0.06, 0.09]	0.07[0.06, 0.09]	0.07[0.06, 0.09]	1.000
CK(U/l), median[IQR]	118.00[84.00, 238.00]	103.00[80.00,181.00]	169.00[107.00,238.00]	0.384
CKMB(U/l), median[IQR]	13.50[7.00, 46.20]	11.30[7.00, 37.50]	14.50[9.33, 61.40]	0.239
LDH(mmol/l), median[IQR]	228.00[195.00, 643.00]	213.00[195.00, 278.00]	253.00[195.00, 665.00]	0.233
ALT(U/l), median[IQR]	25.00[21.00, 40.00]	23.00[21.00, 25.00]	33.00[24.00, 55.83]	0.024
Lpa(mg/l), median[IQR]	155.42[98.83, 313.20]	156.00[123.38, 313.20]	113.80[75.29, 298.34]	0.262
CHOL(mmol/l), mean[IQR]	4.46[3.55, 4.96]	4.47[4.20,4.82]	4.16[3.48,4.97]	0.426
TG(mmol/l), median[IQR]	1.40[1.02, 2.33]	1.31[1.02, 1.77]	1.40[1.19, 2.33]	0.828
HDL(mmol/l), median[±SD]	1.12±0.33	1.16±0.37	1.08±0.29	0.488
LDL(mmol/l), mean(±SD)	2.63±0.76	2.66±0.72	2.60±0.79	0.818
APOA(g/l), median[±SD]	1.28±0.39	1.33±0.36	1.24±0.40	0.519
APOB(g/l), mean(IQR)	0.98[0.82, 1.12]	0.97[0.87, 1.12]	0.98[0.80, 1.12]	0.575
APOA/APOB, median[(±SD]	1.38±0.51	1.43±0.49	1.34±0.52	0.624
NE#, median[(±SD]	4.86±2.23	3.97±1.04	5.60±2.65	0.029
NE%, mean(±SD)	61.46±10.08	59.57±8.88	63.02±10.73	0.343
LYM#, median[±SD]	2.048±0.723	1.970±0.74	2.11±0.70	0.585
LYM%, mean(±SD)	29.04±10.58	29.61±8.46	28.56±12.04	0.784
MON#, median[IQR]	0.50[0.34, 0.64]	0.37[0.30, 0.60]	0.53[0.36, 0.65]	0.255
MON%, mean(IQR)	6.80[5.40, 7.70]	6.80[5.70,7.30]	6.70[4.80,8.05]	0.899
EO#, median[IQR]	0.16[0.05,0.27]	0.16[0.07,0.21]	0.17[0.04,0.29]	1.000
EO%, median[IQR]	2.20[0.90,3.40]	2.50[1.10,3.40]	1.90[0.60,3.20]	0.587
AO(mm), mean(±SD)	28.94±3.66	28.87±3.98	29.00±3.34	0.921
LA(mm), median[IQR]	35.38±6.35	33.20±6.06	37.29±5.98	0.073
RA(mm), mean(±SD)	32.00[31.00, 35.00]	32.00[32.00, 35.00]	32.00[31.00, 35.00]	0.863
LVD(mm), median[(±SD]	48.38±5.60	47.20±4.68	49.41±6.12	0.280
LVS(mm), median[(±SD]	33.13±5.41	30.73±4.92	35.24±4.92	0.018
EF%, median[(±SD]	58.94±11.17	64.47±6.52	54.33±12.10	0.008
MVE ,mean(±SD)	0.79±0.33	0.88±0.35	0.72±0.30	0.247
MVA, mean(±SD)	0.93±0.24	1.00±0.17	0.87±0.26	0.207
EA, median[IQR]	0.74[0.59,1.08]	0.79[0.63,1.21]	0.69[0.58, 0.83]	0.429
FS%, mean(±SD)	31.36±7.28	34.00±5.14	29.17±8.03	0.133
MYO（μg/l), median[IQR]	43.16[30.00, 75.29]	30.00[30.00, 55.26]	59.60[33.23,120.97]	0.023
AST(U/l), median[IQR]	28.00[23.00, 73.00]	24.00[22.00, 36.00]	44.00[26.00, 147.00]	0.089
CTnI(ng/l), median[IQR]	0.02[0.01, 6.33]	0.01[0.01, 0.29]	0.06[0.01, 6.33]	0.181
NT-BNP(g/l), median[IQR]	756.00[119.00, 1236.00]	215.00[50.00, 756.00]	1182.00[383.00, 2420.00]	0.014

Continuous variables are described by mean and SD or median and first–third quartile (interquartile range, IQR), categorical ones by absolute and relative frequencies. FBG, Fasting blood glucose; BUN, Blood Urea Nitrogen; CREA, Creatinine; CK, Creatine kinase; CKMB, Cardiac isoenzyme of creatine kinase; LDH, Lactate dehydrogenase; ALT, Alanine aminotransferase; Lp(a), Lipoprotein a; CHOL, Cholesterol; TG, Triglyceride; HDL, High density lipoprotein; LDL, Low Density Lipoprotein; APOA, Apolipoprotein A; APOB, Apolipoprotein B; NE, Neutrophil; LYM, Absolute lymphocyte count; MON Monocyte; EO, Eosinophil; FDP, Fibrinogen degradation products; AO, Aorta diameter; LA, Left atrial diameter; RA, Right atrial diameter; LVD, left ventricular diameter; LVS, Left ventricular end-systolic diameter; EF%, Ejection fraction; MYO, myoglobin; AST, Aspartate aminotransferase; cTnI, Cardiac troponin I; BNP, Brain natriuretic peptide

Table S2. Characteristics of wild type, αMHC^{Cre+} and aging rats

Variate	Overview (n=18)	WT 12 weeks (n=6)	αMHC^{Cre+} 12 weeks (n=6)	WT 96 weeks (n=6)	p-Value
EF(%), median[IQR]	91.16[79.22, 92.86]	91.90[91.59, 95.23]	91.58[90.12, 93.72]	73.19[69.29, 79.22]	0.003
FS(%), median[SD]	60.21(10.68)	67.42(3.50)	66.31(2.93)	46.90(7.42)	<0.001
LVESD(mm), mean(SD)	2.71(0.95)	1.86(0.34)	2.46(0.62)	3.80(0.48)	<0.001
LVEDD(mm), median[IQR]	6.70[5.54,7.26]	5.48[5.41,6.12]	6.45[5.54,6.85]	7.31[6.78,7.46]	0.013
LVEDV(μl), median[IQR]	231.63[149.92, 268.70]	146.36[141.59, 188.40]	212.540[149.92, 243.65]	281.62[237.83, 294.40]	0.013
LVESV(μl), mean(SD)	33.37(25.91)	11.83(4.93)	24.45(14.78)	63.83(17.30)	<0.001
SVI, mean(SD)	183.13(46.18)	159.23(50.74)	182.51(39.87)	207.64(32.58)	0.219
CO(ml/min), mean(SD)	60.11(13.93)	53.04(14.23)	61.63(11.10)	65.64(13.19)	0.316
LVMass(mg), mean(SD)	975.69(183.60)	928.81(135.98)	1091.19(224.01)	907.06(110.51)	0.187
LVMassCor(mg), mean(SD)	779.72(146.89)	743.05(108.79)	870.45(180.50)	725.65(88.41)	0.200
LVAWs(mm), median[SD]	3.40(0.58)	3.81(0.43)	3.58(0.33)	2.80(0.39)	0.002
LVAWd(mm), median[IQR]	1.88[1.72,2.12]	2.12[1.84,2.52]	1.89[1.88,2.02]	1.68[1.66,1.75]	0.069
LVPWs(mm), mean(IQR)	3.41[3.11,3.72]	3.47[3.41,3.59]	3.76[3.33,4.73]	3.04[2.56,3.15]	0.015
LVPWd(mm), mean(IQR)	2.01[1.81,2.30]	2.02[1.91,2.30]	2.19[2.01,3.54]	1.62[1.61,1.92]	0.078
RBG(mmol/L), Median (SD)	7.64(1.55)	7.17(1.60)	7.60(1.29)	8.15(1.59)	0.59
Weight(g), Median (SD)	292.93(32.08)	274.08(10.67)	275.31(11.34)	329.40(29.14)	<0.001

WT, wild type; EF, ejection fraction; FS, fractional shortening; LVESD, Left Ventricular End - Systolic Dimension; LVEDD, Left Ventricular End - Diastolic Dimension; LVEDV, Left Ventricular End - Diastolic Volume; LVESV, Left Ventricular End - Systolic Volume; SVI, Stroke Volume Index; CO,cardiac output; LVMass,left ventricular mass; LVMass cor Left Ventricular Mass Corrected; LVAWs,Left Ventricular Anterior Wall Thickness in Systole; LVAWd,Left Ventricular Anterior Wall Thickness in Diastole; LVPWs, Left Ventricular Posterior Wall Thickness in Systole; LVPWd, Left Ventricular Posterior Wall Thickness in Diastole; RBG, random blood glucose.

Table S3. Characteristics of T1DM and control rats

Variate	Overview (n=10)	Control (n=5)	T1DM (n=5)	p-Value
EF% ,mean(SD)	74.51(16.03)	90.23(0.96)	58.79(4.272)	<0.001
FS% ,median[IQR]	37.22[30.59,61.93]	61.93[61.47,62.74]	30.588[30.467,31.052]	0.008
LVESD(mm), mean(SD)	3.09(1.04)	2.22(0.49)	3.95(0.66)	0.003
LVEDD(mm), mean(SD)	5.81(1.03)	5.85(1.20)	5.77(0.83)	0.910
LVESV(μl), mean(SD)	44.67(33.46)	18.26(8.99)	71.08(27.64)	0.007
LVEDV(μl), mean(SD)	174.74(68.62)	180.18(77.95)	169.30(57.31)	0.828
SVl, mean(SD)	130.07(62.09)	161.92(68.97)	98.22(30.40)	0.129
CO(ml/min), mean(SD)	34.50(15.99)	45.02(15.30)	23.97(7.49)	0.039
LVMass(mg), mean(SD)	760.17(215.37)	697.22(183.68)	823.12(226.06)	0.412
LVMassCor(mg), mean(SD)	608.13(172.29)	557.77(146.95)	658.49(180.84)	0.412
LVAWs(mm), median[IQR]	2.80[2.15,2.86]	2.83[2.81,2.89]	2.15[1.81,2.80]	0.095
LVAWd(mm), mean(SD)	1.74(0.52)	1.51(0.37)	1.96(0.54)	0.208
LVPWs(mm), mean(SD)	3.06(0.63)	3.55(0.45)	2.57(0.31)	0.007
LVPWd(mm), mean(SD)	2.16(0.62)	2.18(0.75)	2.14(0.43)	0.917
6w FBG(mmol/L), mean(SD)	4.87(0.74)	4.96(0.54)	4.78(0.88)	0.737
6w Weight(g), mean(SD)	257.80(19.48)	270.00(18.97)	245.60(10.07)	0.053
10w FBG(mmol/L), median[IQR]	6.70[6.10,33.30]	6.10[5.70,6.40]	33.30[29.60,33.30]	0.011
10w Weight(g), mean(SD)	259.01(28.89)	281.22(4.41)	236.80(25.76)	0.009
18w FBG(mmol/L), mean(SD)	17.63(12.81)	5.12(0.12)	30.14(3.92)	<0.001
18w Weight(g), mean(SD)	254.70(68.39)	317.80(13.23)	191.60(34.86)	<0.001

T1DM, type 1 diabetes mellitus; EF, ejection fraction; FS, fractional shortening; LVESD, Left Ventricular End - Systolic Dimension; LVEDD, Left Ventricular End - Diastolic Dimension; LVEDV, Left Ventricular End - Diastolic Volume; LVESV, Left Ventricular End - Systolic Volume; SVl, Stroke Volume Index; CO,cardiac output; LVMass,left ventricular mass; LVMass cor Left Ventricular Mass Corrected; LVAWs,Left Ventricular Anterior Wall Thickness in Systole; LVAWd,Left Ventricular Anterior Wall Thickness in Diastole; LVPWs, Left Ventricular Posterior Wall Thickness in Systole; LVPWd, Left Ventricular Posterior Wall Thickness in Diastole; FBG,Fasting blood glucose

Table S4. Characteristics of T2DM and control rats with or without *Rnd3* gene intervening

Variate	Overview (n=36)	WT_Control (n=6)	WT_T2DM (n=6)	OE_Control (n=6)	OE_T2DM (n=6)	KO_Control (n=6)	KO_T2DM (n=6)	p-Value
EF(%), median [SD]	85.35 (10.21)	91.86 (1.97)	81.08 (7.10)	94.38 (2.44)	89.02 (5.30)	87.89 (3.42)	67.91 (7.73)	<0.001
FS(%), median [SD]	57.94 (11.88)	64.89 (3.90)	51.54 (7.41)	70.84 (5.13)	61.72 (7.31)	59.51 (5.53)	39.12 (6.33)	<0.001
LVESD(mm) ,mean(IQR)	2.49[1.94, 3.01]	2.04[2.03, 2.21]	2.92[2.69, 3.35]	1.76[1.37, 1.79]	1.94[1.82, 3.01]	2.47[2.40, 2.69]	3.38[3.03, 4.47]	<0.001
LVEDD(mm), median[SD]	6.07 (0.54)	5.83 (0.34)	6.35 (0.42)	5.81 (0.58)	6.17 (0.48)	6.08 (0.33)	6.19 (0.72)	0.48
LVESV(μl), mean(IQR)	22.16[13.18, 36.03]	13.84[13.42, 16.47]	32.75[26.71, 46.38]	9.26[4.82, 10.94]	13.18[10.14, 36.28]	21.76[20.30, 27.45]	46.98[36.03, 91.34]	<0.001
LVEDV(μl), median[SD]	187.32 (38.00)	169.78 (23.68)	206.69 (31.79)	169.75 (36.92)	193.62 (33.23)	186.71 (23.30)	197.35 (54.31)	0.49
SVI, mean (SD)	158.57 (30.94)	156.03 (22.93)	168.65 (34.36)	160.26 (34.92)	170.98 (23.03)	164.36 (23.28)	131.15 (26.34)	0.27
CO(ml/min), mean(IQR)	52.13(41.64, 66.23)	50.85(41.64, 61.56)	66.23[50.69, 70.45]	59.05[41.89, 69.73]	52.17[37.56, 64.54]	47.51[44.90, 67.31]	39.40[38.31, 44.32]	0.19
LVMass(mg), mean(SD)	931.68 (242.92)	963.34 (280.41)	949.75 (164.03)	890.34 (247.17)	777.80 (133.55)	970.74 (158.78)	1038.12 (323.76)	0.59
LVMassCor(mg), mean(SD)	745.35 (194.34)	770.67 (224.33)	759.80 (131.23)	712.27 (197.73)	622.24 (106.84)	776.60 (127.02)	830.49 (259.01)	0.59
LVAWs(mm), median[SD]	3.29 (0.47)	3.47 (0.40)	3.02 (0.38)	3.59 (0.40)	3.43 (0.21)	3.38 (0.45)	2.85 (0.43)	0.04
LVAWd(mm), median[SD]	1.94 (0.29)	1.96 (0.31)	1.79 (0.27)	2.16 (0.24)	1.82 (0.21)	1.96 (0.27)	1.95 (0.26)	0.32
LVPWs(mm), mean(SD)	3.61 (0.57)	4.07 (0.52)	3.59 (0.40)	4.00 (0.63)	3.31 (0.15)	3.53 (0.31)	3.17 (0.61)	0.03
LVPWd(mm), mean(SD)	2.29 (0.49)	2.50 (0.59)	2.34 (0.23)	2.11 (0.31)	1.91 (0.13)	2.41 (0.33)	2.49 (0.73)	0.23
6w FBG (mmol/L), mean(SD)	8.46 (2.11)	9.65 (2.95)	8.52 (2.14)	8.42 (2.63)	8.82 (0.89)	7.60 (1.73)	7.77 (1.98)	0.628
6w Weight (g), mean (SD)	150.00 (16.72)	151.30 (11.11)	169.20 (16.90)	134.70 (5.39)	140.70 (17.96)	157.30 (10.93)	146.80 (13.01)	0.006
10w FBG (mmol/L), mean(SD)	17.37 (9.01)	9.32 (1.81)	26.45 (3.61)	8.70 (2.52)	24.35 (3.48)	8.50 (1.52)	26.92 (1.25)	<0.001
10w Weight (g), mean (SD)	302.90 (47.65)	290.30 (18.90)	310.70 (12.68)	236.70 (52.88)	362.70 (62.37)	313.00 (19.85)	340.20 (17.34)	0.001
18w FBG (mmol/L), median[IQR]	16.11 (8.45, 30.15)	8.60 (8.15, 8.83)	32.40(30.20, 33.25)	6.50(6.00, 12.55)	24.75(22.05, 30.53)	8.10(6.58, 10.45)	30.30(28.80, 32.63)	<0.001
18w Weight (g), median [IQR]	355.50(323.55, 383.50)	355.50(343.50, 393.38)	331.90(320.25, 345.20	358.25(332.55, 367.38)	305.45(292.75, 312.98	367.50(356.25, 372.00)	393.50(386.25, 399.25)	<0.001
18w Length of tibia (cm), mean(IQR)	5.50 (5.00, 5.80)	5.65 (5.50, 5.95)	3.50 (3.35, 3.65)	5.35 (5.05, 5.88)	5.35 (5.05, 5.58)	5.55 (5.50, 5.68)	5.85 (5.80, 5.98)	<0.001
18w Heart weight (g), mean(IQR)	1.60 (1.38, 1.73)	1.65 (1.28, 1.80)	1.85 (1.65, 1.90)	1.55 (1.28, 1.60)	1.40 (1.30, 1.58)	1.40 (1.25, 1.40)	1.70 (1.70, 1.78)	0.018

Continuous variables are described by mean and SD or median and first–third quartile (interquartile range, IQR). T2DM, type 2 diabetes mellitus; WT, wild type; EF, ejection fraction; FS, fractional shortening; LVESD, Left Ventricular End - Systolic Dimension; LVEDD, Left Ventricular End - Diastolic Dimension; LVEDV, Left Ventricular End - Diastolic Volume; LVESV, Left Ventricular End - Systolic Volume; SVI, Stroke Volume Index; CO,cardiac output; LVMass,left ventricular mass; LVMass cor Left Ventricular Mass Corrected; LVAWs,Left Ventricular Anterior Wall Thickness in Systole; LVAWd,Left Ventricular Anterior Wall Thickness in Diastole; LVPWs, Left Ventricular Posterior Wall Thickness in Systole; LVPWd, Left Ventricular Posterior Wall Thickness in Diastole; FBG,Fasting blood glucose

Table S5. Differential expression of miRNAs enriched by miRNA sequencing

Gene ID	log2(HG/NG)	P_value (HG/NG)	Q_value (HG/NG)
hsa-miR-101-5p	1.45	5.13E-03	2.84E-02
hsa-miR-103a-3p	5.51	0.00E+00	0.00E+00
hsa-miR-103b	-1.59	0.00E+00	0.00E+00
hsa-miR-107	1.05	1.94E-10	2.22E-09
hsa-miR-1273h-5p	-2.73	2.56E-03	1.54E-02
hsa-miR-1973	-1.87	2.11E-21	3.59E-20
hsa-miR-199b-3p	-5.85	0.00E+00	0.00E+00
hsa-miR-200a-3p	1.89	4.53E-04	3.25E-03
hsa-miR-3163	-3.29	3.54E-03	2.03E-02
hsa-miR-3194-3p	1.45	5.63E-07	5.40E-06
hsa-miR-33b-5p	-1.38	7.88E-07	7.51E-06
hsa-miR-3529-3p	8.49	0.00E+00	0.00E+00
hsa-miR-365a-3p	-4.75	5.70E-64	2.16E-62
hsa-miR-3691-5p	1.90	2.12E-03	1.30E-02
hsa-miR-3913-5p	1.72	8.69E-03	4.40E-02
hsa-miR-423-5p	-1.05	0.00E+00	0.00E+00
hsa-miR-4463	-1.61	2.41E-05	1.97E-04
hsa-miR-4484	1.05	2.09E-04	1.54E-03
hsa-miR-4485-5p	-2.24	1.01E-03	6.73E-03
hsa-miR-4689	-3.47	1.09E-05	9.23E-05
hsa-miR-4732-3p	3.24	1.30E-21	2.23E-20
hsa-miR-4804-5p	-1.74	1.47E-03	9.42E-03
hsa-miR-5090	2.24	6.10E-03	3.28E-02
hsa-miR-548ad-5p	-5.12	3.32E-34	7.72E-33
hsa-miR-548ae-5p	-1.75	9.00E-08	8.89E-07
hsa-miR-548ap-3p	2.20	1.40E-22	2.48E-21
hsa-miR-548o-5p	-7.43	7.64E-04	5.23E-03
hsa-miR-6511a-3p	1.23	1.17E-04	9.06E-04
hsa-miR-6820-3p	1.31	2.36E-03	1.43E-02
hsa-miR-7-5p	-8.57	0.00E+00	0.00E+00
hsa-miR-7974	1.15	3.79E-15	5.03E-14
hsa-miR-937-5p	1.20	8.95E-04	6.03E-03
novel-hsa-miR16-3p	1.21	7.08E-03	3.69E-02
novel-hsa-miR184-3p	-2.37	4.95E-06	4.48E-05
novel-hsa-miR187-5p	3.55	1.16E-03	7.61E-03
novel-hsa-miR193-5p	-1.40	1.31E-04	1.00E-03
novel-hsa-miR243-5p	1.44	1.02E-05	8.63E-05
novel-hsa-miR263-3p	-3.29	3.54E-03	2.03E-02
novel-hsa-miR295-3p	1.63	5.47E-04	3.86E-03
novel-hsa-miR300-3p	1.16	7.07E-06	6.27E-05
novel-hsa-miR35-5p	-1.04	1.36E-04	1.04E-03
novel-hsa-miR56-5p	1.20	8.43E-03	4.28E-02
novel-hsa-miR99-5p	2.56	5.47E-03	2.99E-02

HG, high glucose; NG, normal glucose

Table S6. Characteristics of clinical cohorts for circulating miR-103a-3p test

Variate	Overview (n=41)	Control (n=17)	Diabetes (n=24)	p-Value
Female, n(%)	14(34.15)	7(41.18)	7(29.17)	0.424
Man, n(%)	27(65.85)	10(58.82)	17(70.83)	
Age, mean(±SD)	62.20±8.24	63.12±9.48	61.54±7.17	0.558
miR-103a-3p, median [IQR]	0.26[0.09,0.58]	0.04[0.02,0.12]	0.54[0.31,0.61]	<0.001
FBG(mmol/L), median[IQR]	5.91[4.76,8.09]	4.92[4.30,5.81]	7.14[5.65,9.38]	<0.001
Tyg, median[IQR]	1.11[0.86,1.29]	0.86[0.77,0.97]	1.28[1.11,1.37]	<0.001
NE#(10^9/L), median[IQR]	4.56[3.52,6.00]	4.78[3.61,6.29]	4.52[3.40,5.35]	0.388
NE%, median[IQR]	62.70[57.30,70.80]	68.00[58.10,74.60]	60.50[54.80,64.20]	0.183
LYM#(10^9/L), mean(±SD)	1.91±0.77	1.72±0.74	2.06±0.75	0.173
LYM%, mean(±SD)	26.73±10.09	23.79±10.66	29.00±8.99	0.115
MON#(10^9/L), mean(±SD)	0.42±0.13	0.42±0.14	0.42±0.13	0.932
MON%, median[IQR]	5.90[4.90,7.00]	5.10[4.70,7.30]	5.90[4.90,7.00]	0.821
EO#(10^9/L), median[IQR]	0.11[0.05,0.18]	0.15[0.05,0.19]	0.09[0.04,0.15]	0.487
EO%, median[IQR]	1.40[0.60,3.10]	2.20[0.70,3.10]	1.20[0.60,2.90]	0.307
ALT(U/L), median[IQR]	21.00[15.00,32.00]	18.00[14.00,30.00]	23.00[20.00,34.00]	0.136
AST(U/L), median[IQR]	21.00[17.00,24.00]	21.00[17.00,24.00]	21.00[17.00,24.00]	0.670
ALP(U/L), median[IQR]	73.00[63.00,86.00]	73.00[68.00,82.00]	74.00[59.00,86.00]	1.000
ALB(g/L), median[IQR]	36.20[34.60,38.90]	35.10[33.40,37.00]	37.20[36.00,39.60]	0.061
TC(mmol/L), median[IQR]	4.65[3.75,5.38]	3.93[3.62,4.65]	5.22[4.18,5.83]	0.039
TG(mmol/L), median[IQR]	1.41[1.05,1.93]	1.19[0.98,1.53]	1.82[1.35,2.10]	0.020
HDL(mmol/L),m ean(±SD)	1.08±0.24	1.15±0.26	1.03±0.22	0.141
LDL(mmol/L), median[IQR]	2.79[1.98,3.67]	2.22[1.98,2.79]	3.51[2.39,3.89]	0.046
Lp(a)(mg/L), mean(±SD)	1.14±0.20	1.17±0.24	1.12±0.17	0.453
APOB(g/L), median[IQR]	0.99[0.77,1.19]	0.90[0.73,1.03]	1.16[0.92,1.32]	0.078
BUN(mmol/L), mean(±SD)	5.40±1.45	5.41±1.61	5.40±1.33	0.974
Cr(μmol/L), median[IQR]	70.50[56.80,83.90]	72.70[61.50,106.00]	70.50[51.50,82.50]	0.367
UA(μmol/L）, median[IQR]	355.00[285.00,417.00]	367.00[285.00,375.00]	355.00[287.00,443.00]	0.636

Continuous variables are described by mean and SD or median and first–third quartile (interquartile range, IQR), categorical ones by absolute and relative frequencies. FBG, Fasting blood glucose; Tyg, Triglyceride-glucose index; ALT, Alanine aminotransferase; AST, Aspartate aminotransferase; ALP, Alkaline phosphatase; ALB, Albumin; TC, Total cholesterol; TG, Triglyceride; HDL, High density lipoprotein; LDL, Low Density Lipoprotein; Lp(a), Lipoprotein a; APOB, Apolipoprotein B; BUN, Blood Urea Nitrogen; Cr, Creatinine; UA, Serum trioxypurine.

Table S7. Characteristics of T2DM and control rats with/without AAV9_miR103a-3p_sponges infusion

Variate	Overview (n=24)	WT_Control (n=6)	WT_T2DM (n=6)	OE_Control (n=6)	OE_T2DM (n=6)	p-Value
EF(%), median[IQR]	90.84[84.08, 96.78]	93.06[90.84, 99.28]	62.74[61.51, 85.67]	96.77[91.85, 99.43]	87.20[84.92, 93.89]	0.013
FS(%), median[IQR]	63.28[53.80, 75.58]	67.62[63.28, 85.37]	34.63[33.77, 56.20]	75.58[64.93, 87.15]	57.40[55.02, 70.82]	0.014
LVESD(mm), mean(SD)	2.08(1.11)	3.37(0.96)	1.49(0.62)	2.06(0.69)	1.42(0.85)	0.003
LVEDD(mm), median[IQR]	5.58(0.62)	5.64(0.77)	5.47(0.60)	5.63(0.39)	5.60(0.65)	0.972
LVESV(μl), mean(IQR)	15.19[4.03, 26.51]	31.59[19.08, 84.38]	7.22[0.78, 13.32]	15.19[10.99, 26.51]	2.69[0.94, 14.99]	0.010
LVEDV(μl), median[SD]	161.07(40.21)	182.37(49.73)	148.51(36.64)	156.78(23.66)	156.61(37.98)	0.538
SVI, mean(IQR)	148.19[106.75, 168.73]	117.38[111.79, 140.54]	106.75[105.02, 178.55]	148.19[103.46, 167.72]	161.29[137.94, 168.97]	0.870
CO(ml/min), mean(SD)	48.55(12.99)	50.42(12.49)	43.81(15.48)	51.33(10.27)	48.62(11.87)	0.790
LVMass(mg), mean(SD)	876.88(219.10)	889.54(223.08)	844.52(243.16)	939.85(196.13)	833.63(193.96)	0.858
LVMassCor(mg), mean(SD)	702.41(176.44)	711.63(178.46)	675.62(194.53)	751.88(156.91)	670.50(161.09)	0.870
LVAWs(mm), median[IQR]	3.46[3.08,3.74]	3.59[3.46,3.78]	2.54[2.18,3.38]	3.71[3.39,3.82]	3.08[3.05,3.72]	0.093
LVAWd(mm), median[IQR]	2.01[1.68,2.30]	2.01[1.99,2.48]	1.73[1.42,2.27]	2.09[1.93,2.30]	1.68[1.65,2.31]	0.611
LVPWs(mm), mean(SD)	3.61(0.88)	4.03(0.82)	3.00(0.57)	3.87(0.38)	3.56(1.15)	0.205
LVPWd(mm), mean(SD)	2.30(0.60)	2.34(0.37)	2.22(0.54)	2.51(0.35)	2.15(0.91)	0.783
6w FBG(mmol/L), Median (IQR)	7.50 (6.80, 9.65)	8.00 (7.18, 9.35)	6.70 (6.35, 7.05)	8.55 (6.65, 10.60)	8.55 (7.38,10.85)	0.306
6w Weight(g), Median (IQR)	178.50 (164.75, 186.00)	175.00 (166.25, 180.75)	181.50 (172.75, 191.00)	170.50 (159.75, 182.00)	183.50 (175.75, 186.00)	0.403
10w FBG (mmol/L), Median (IQR)	15.65 (9.30, 27.05)	9.00 (8.53, 9.63)	28.60 (24.43, 30.90)	9.30 (8.78, 9.68)	26.70 (24.85, 27.95)	<0.001
10w Weightg(g), Median (IQR)	260.50 (232.25, 300.50)	216.00 (213.25, 232.25)	249.50 (238.00, 259.50)	268.00 (261.75, 308.75)	302.00 (285.50, 358.25)	0.045
18w FBG (mmol/L), Median[IQR]	15.65 (9.30, 26.45)	8.75 (6.08, 10.38)	27.95 (21.40, 33.30)	9.30 (8.70, 9.68)	27.00 (24.85, 28.70)	<0.001
18w Weight(g), Median (IQR)	299.85 (288.08, 362.08)	288.95 (270.98, 296.95)	298.65 (289.13, 310.80)	333.20 (296.48, 379.68)	377.15 (298.40, 454.55)	0.29
18w Length of tibia (cm), Median (IQR)	4.95 (4.48, 5.11)	4.86 (4.38, 5.25)	4.95 (4.68, 5.08)	5.00 (4.60, 5.10)	4.80 (4.45, 5.08)	>0.999
18w Heart weight (g), Median (IQR)	1.56 (1.33, 1.70)	1.29 (1.20, 1.36)	1.780 (1.76, 1.83)	1.57 (1.53, 1.63)	1.35 (1.08, 1.53)	0.01

T2DM, type 2 diabetes mellitus; EF, ejection fraction; FS, fractional shortening; LVESD, Left Ventricular End - Systolic Dimension; LVEDD, Left Ventricular End - Diastolic Dimension; LVEDV, Left Ventricular End - Diastolic Volume; LVESV, Left Ventricular End - Systolic Volume; SVI, Stroke Volume Index; CO,cardiac output; LVMass,left ventricular mass; LVMass cor Left Ventricular Mass Corrected; LVAWs,Left Ventricular Anterior Wall Thickness in Systole; LVAWd,Left Ventricular Anterior Wall Thickness in Diastole; LVPWs, Left Ventricular Posterior Wall Thickness in Systole; LVPWd, Left Ventricular Posterior Wall Thickness in Diastole; FBG,Fasting blood glucose

Table S8. Primer sequences for quantitative real-time PCR

Gene	Specie	Forward primer，5'-3'	Reversed primer，5'-3'
β-actin	Rat	CCTGTATGCCTCTGGTCGT	CTGTAGCCACGCTCGGT
Rnd3	Rat	GCAAGAGCAAACGGAAAG	CATGCCGAAACTAAGGACA
miR-103a-3p	Rat	GGAGCAGCATTGTACAGGG	CACACTCTCACTCACGCATC
IL-6	Rat	ATTGTATGAACAGCGATGATGCAC	CCAGGTAGAAACGGAACTCCAGA
MCP1	Rat	GGGCCAGAGGATTGTCCAAAT	TGTCTCCCCTCAGTACCGTC
IL-1α	Rat	GGCACAGAGGGAGTCAACTCAT	ATCTGGGTTGGATGGTCTCTTCTAA
P53	Rat	CAGGGAGTGCAAAGAGAGCA	TCTCGGAACATCTCGAAGCG
P16	Rat	TTCACCAAACGCCCCGAA	TCGAATCTGCACCATAGGAGA
GDF15	Rat	CTCAGAACCAACCCCTGACC	TAGGCTTCGGGGAGACCC
Rnd3	Human	AATAGAGTTGAGCCTGTGGG	CTAATGTACTAACATCTGTCCGC
miR-103a-3p	Human	GGAGCAGCATTGTACAGGG	TTGGGAGGTAGGAGGTTGAT
β-actin	Human	TCTCCCAAGTCCACACAGG	GGCACGAAGGCTCATCA
